# Tyrosinase Depletion Prevents the Maturation of Melanosomes in the Mouse Hair Follicle

**DOI:** 10.1371/journal.pone.0143702

**Published:** 2015-11-30

**Authors:** Elyse K. Paterson, Thomas J. Fielder, Grant R. MacGregor, Shosuke Ito, Kazumasa Wakamatsu, Daniel L. Gillen, Victoria Eby, Raymond E. Boissy, Anand K. Ganesan

**Affiliations:** 1 Department of Biological Chemistry, University of California Irvine, Irvine, CA, United States of America; 2 University of California Irvine Transgenic Mouse Facility, University Laboratory Animal Resources, Office of Research, Irvine, CA, United States of America; 3 Department of Developmental and Cell Biology, University of California Irvine, Irvine, CA, United States of America; 4 Department of Chemistry, Fujita Health University School of Health Sciences, Toyoake, Aichi 470–1192, Japan; 5 Department of Statistics, University of California Irvine, Irvine, CA, United States of America; 6 Department of Dermatology, University of Cincinnati, Cincinnati, OH, United States of America; 7 Department of Dermatology, University of California Irvine, Irvine, CA, United States of America; Wenzhou Medical University, CHINA

## Abstract

The mechanisms that lead to variation in human skin and hair color are not fully understood. To better understand the molecular control of skin and hair color variation, we modulated the expression of *Tyrosinase (Tyr)*, which controls the rate-limiting step of melanogenesis, by expressing a single-copy, tetracycline-inducible shRNA against *Tyr* in mice. Moderate depletion of TYR was sufficient to alter the appearance of the mouse coat in black, agouti, and yellow coat color backgrounds, even though TYR depletion did not significantly inhibit accumulation of melanin within the mouse hair. Ultra-structural studies revealed that the reduction of *Tyr* inhibited the accumulation of terminal melanosomes, and inhibited the expression of genes that regulate melanogenesis. These results indicate that color in skin and hair is determined not only by the total amount of melanin within the hair, but also by the relative accumulation of mature melanosomes.

## Introduction

Skin color varies widely both within and between human ethnic populations, evolving over generations to be darker in indigenous equatorial populations to protect the skin from UV damage [1, 2], or to be lighter in populations at higher latitudes to facilitate Vitamin D production [3]. With human civilization, lighter skinned populations have moved to more temperate climates, resulting in the increased incidence and prevalence of UV-induced skin cancer. Indeed, one in six Americans [4] and one in two Australians will develop skin cancer during their lifetime [5]. Understanding the molecular mechanisms of skin color heterogeneity could lead to the development of new strategies to prevent skin cancer in skin types that are more susceptible to UV-induced damage.

Numerous proteins have been identified that confer differences in coat color between inbred animal strains [6–8]. Despite this information, it is not yet fully understood how variation in individual pigment-related genes results in the diversity of skin color phenotypes observed in nature. Melanin is a chemically inert yet stable pigment that gives skin and hair its color [9]. The two primary melanins found in human hair and skin are the red/yellow pheomelanins and the brown/black eumelanins [9]. Melanins are synthesized from tyrosine via an enzymatic reaction catalyzed by tyrosinase (TYR) [10, 11] with tyrosinase-related protein 1 (TYRP1) and DOPAchrome tautomerase (DCT) also being required to generate the final melanin product [10]. TYR is a membrane glycoprotein that catalyzes the conversion of tyrosine to DOPA [12] and then subsequently oxidizes DOPA to form DOPAquinone. This intermediate is further acted upon by TYRP1 and DCT to form eumelanin [9, 12]. TYRP1 and DCT are also involved in the proper trafficking of TYR to the stage II melanosome and in detoxification processes in the melanosome, respectively [13, 14]

Melanin synthesis occurs within the melanosome, a specific lysosome-related organelle that matures through four morphologic stages (I-IV) [15–19]. Stage I melanosomes are spherical vacuoles that lack TYR activity and melanin. They contain intralumenal fibrils that are comprised mainly of luminal fragments of PMEL5/gp100, an integral membrane protein specifically expressed in pigment-producing cells [9, 16, 18, 20, 21]. In the stage II melanosome, PMEL5 is organized into sheets and thus transforms the spherical stage I melanosome to an elongated, fibrillar organelle [12, 20, 22]. TYR is transported to the stage II melanosome, initiates melanin synthesis, and deposits pigment onto internal fibrils that are characteristic of the stage III melanosome [15, 16, 18, 20]. Stage IV melanosomes are either elliptical or ellipsoidal in shape and demonstrate complete melanization with little TYR enzymatic activity [15, 18]. Stage IV melanosomes are defined by the absence of visible amyloid fibrils [23, 24]. Mature, stage IV melanosomes are transferred from melanocytes to adjacent keratinocytes where they accumulate as melanin caps above the keratinocyte nuclei and absorb disruptive UV-radiation before it can damage the DNA [25]. Correlative studies have identified biochemical and ultra-structural alterations thought to be responsible for skin and hair color variation. While light and dark skinned individuals possess similar numbers of melanocytes, melanosomes are larger (0.5–0.8 μm dia.) in highly pigmented skin compared to lightly pigmented skin (0.3–0.5 μm dia.) [26–31]. Furthermore, lightly pigmented skin contains less dense melanosomes, mostly at stage II and III, while darkly pigmented skin contains denser melanosomes, mostly at stage IV [26–31]. Melanocytes from light skinned individuals also synthesize TYR protein more slowly, degrade TYR at a faster rate, and contain less TYR activity when compared to melanocytes from dark-skinned individuals [32]. Recent RNAi-based functional genomics studies have identified a large number of novel genes that regulate melanogenesis by controlling the expression and stability of TYR [33]. Taken together, these studies suggest that subtle variations in *Tyr* expression and activity may contribute to the diversity seen in human skin color. We tested this prediction experimentally using a novel inducible and reversible partial *Tyr* loss of function mouse model. We demonstrate that partial depletion of TYR alters mouse coat color, inhibits normal melanosome maturation and inhibits expression of genes that regulate melanogenesis, while only subtly affecting eumelanin accumulation. These results support a model where TYR is necessary not only for the synthesis of melanin, but also for the complete maturation of the stage IV melanosome, phenotypes which could have only been appreciated in a partial loss of function model.

## Materials and Methods

### Cell Lines and Reagents

B16F1 mouse melanoma cells (a gift from William Pavan, NHGRI obtained from ATCC) were cultured in high-glucose DMEM media supplemented with 10% FBS, 2mM glutamine, 1x NEAA, and 0.075% sodium bicarbonate at 5% CO_2_ in air. The pCol-TGM plasmid was kindly provided by Scott Lowe. The pCAGS-Flpe-puro plasmid was purchased from Addgene (Cambridge, MA). The *Tyr*-shRNAs (*Tyr*-shRNA #1 and *Tyr*-shRNA #2) were purchased from Open Biosystems/GE Dharmacon (Lafayette, CO) in the pGIPZ plasmid. The 5’ to 3’mature antisense sequence for *Tyr*-shRNA #1 is: TCTTCTGAAGGCATAGCCT, while that for *Tyr*-shRNA #2 is: GATCTGCTACAAATGATCT. Antibodies used in this study are listed in S2 Table. All of the primers used for genotyping the mice were taken from [34], the mouse mutant resource website, (Jackson Laboratory Bar Harbor, ME) or designed using Primer3web (http://www.primer3plus.com). Oligodeoxynucleotide primers (Eurofins Scientific, Huntsville, AL) are listed in S3 Table.

### Identification and characterization of a potent shRNA against *Tyr*

Short-hairpin RNAs targeting *Tyr* were analyzed using a “Sensor Rules” algorithm to identify highly potent shRNAs effective at the single copy level in the cell [35]. Two shRNAs were chosen for further analysis because they fulfilled all but one of the selected criteria of the algorithm. To generate lentiviral plasmids containing both shRNAs, TLA-HEK293T cells (Thermo-Scientific/Open Biosystems, Grand Island, NY) were seeded at a density of 3.8 x 10^5^ cells per well of a 12-well plate and transfected with 1.4 μg of pCMV packaging vector (Thermo Scientific), 0.5 μg pMP2G packaging vector (Thermo Scientific), and 2.0 μg of pGIPZ-*Tyr* or pGIPZ-*NT* (non-targeting control) (Open Biosystems/GE Dharmacon) for a total of 3.9 μg of DNA. Arrest-In was used as the transfection reagent at a 1:5 DNA:Arrest-In ratio (Thermo Scientific/Open Biosystems). Lentiviral supernatant was harvested at 48 and 72 hours post-transfection and filtered through a 0.45μm filter to remove cells. Lentivirus quantitation was carried out using a Lentivirus-Associated p24 ELISA kit (Cell Biolabs, Inc, San Diego, CA) according to the manufacturer’s instructions. B16 mouse melanoma cells were infected in the presence of Polybrene (8μg/ ml) (Santa Cruz Biotechnology, Santa Cruz, CA) with lentivirus containing either pGIPZ-*Tyr* or pGIPZ-*NT* at multiplicity of infection (MOI) of 0.1. Infected B16 cells were selected for using 2 μg/mL of puromycin (Acros Organics, Pittsburgh, PA), and expanded in culture. To quantitate knockdown of Tyr mRNA and protein, infected cells were harvested for quantitative RT-PCR and western blot analysis as previously described [36].

### Knock-in of a doxycycline-inducible shRNA targeting *Tyr*

KH2 cells were cultured on mitotically inactivated primary CD-1 mouse embryonic fibroblasts as described [37]. Short-hairpin RNAs were cloned into the pCol1a1 FLP-in vector [34, 38]. The shRNA was integrated immediately downstream of the *Col1a1* locus via FLPe-mediated homologous recombination (HR) via the single Frt site integrated at the mouse *Col1a1* locus [37]. Approximately 2 x 10^7^ KH2 ES cells were electroporated with a mixture of 20 μg of the shRNA-containing vector and 10 μg of pCAGS-FLPe-puro (Addgene) [39]. Selection for HR was performed using hygromycin (150 μg/ml) beginning 48 hours post-electroporation. Correct targeting of the *Col1a1* locus in KH2 cells with the pCol-TGM vector reconstitutes the start codon for an existing hygromycin expression cassette. Sixteen independent hygromycin resistant clones were picked and expanded for Southern analysis. Targeted cells were trypsinized to single cell suspension and injected into C57BL/6NTac blastocysts and surviving embryos implanted into pseudo-pregnant ICR foster moms. Chimeric offspring were identified by agouti coat color.

### Generation of *Tyr*-shRNA mutant mice on a variety of coat-color and transactivator backgrounds

All experiments involving mice conform to the Guide for the Care and Use of Laboratory Animals, 8th edition (National Academies Press, Washington, D.C.) and were approved by the Institutional Animal Care and Use Committee (IACUC) of the University of California, Irvine, approval number 2011–3020. Mice were anesthetized via injection with a sterile sodium chloride solution of 10 mg/mL ketamine and 1.25 mg/mL xylazine solution in the lower ventral area. Mice were euthanized by inhalation of carbon dioxide followed by exsanguination. Mice were housed in our approved animal facility with 12-hour light cycles. Food and water were provided ad libitum. Animals were monitored daily, and any mice exhibiting signs of distress were euthanized. The KH2 ES cell line was derived from a 129S4 x C57BL/6J F1 embryo. Four month old male ES cell chimeras, confirmed by genotyping to contain the *Tyr*-shRNA and the reverse tetracycline transactivator rtTA2, were crossed with four-month-old C57BL/6J females (stock number 000664, Jackson Laboratory, Bar Harbor, ME) to produce 129S4; C57BL/6J N2 progeny with the following genotypes: wild-type, *Tyr*-shRNA alone, rtTA2 alone, or *Tyr*-shRNA and rtTA2 together. These animals were then used to introduce the *Tyr*-shRNA/R26-rtTA2 transgenes onto three different coat color backgrounds (i.e. white-bellied agouti (*A* ^*w*^), yellow agouti (*A* ^*y*^), or non-agouti (*a/a*, black) using the following breeding strategies. Non-agouti 129S4; C57BL/6J N2 mice containing *Tyr*-shRNA and rtTA2 were bred with white-bellied agouti 129S1/SvImJ (Jackson Lab, stock number 002448) mice or yellow-agouti B6.Cg-A^y^/J mice (Jackson Laboratory, stock number 000021). Agouti (*A* ^*w*^ */+*or *A* ^*y*^ */+*) progeny containing the appropriate transgenes were intercrossed for four generations to generate progeny with an agouti (*A* ^*w*^, or *A* ^*y*^) coat color and *Tyr*-shRNA and rtTA2 transgenes having the following overall strain background composition; 37.5% B6J, 12.5% 129S4, 50% 129S1 (for animals derived from crosses with 129S1/ImJ); or 87.5% B6J, 12.5% 129S4 for animals derived from crosses with B6.Cg-A^y^/J mice. Transgenic mice positive for both *Tyr*-shRNA and CAG-rtTA3 were generated by crossing agouti C57BL/6J N2 progeny containing only the *Tyr*-shRNA transgene with B6N.FVB(Cg)-Tg(CAG-rtTA3)4288Slowe/J mice (Jackson Laboratory, stock number 016532). Non-agouti *Tyr*-shRNA and CAG-rtTA3 positive progeny from this mating (essentially N3 for B6) were then crossed with C57BL/6J mice, and offspring (essentially N4 for B6) containing both rTTA3 and Tyr-shRNA transgenes were intercrossed for four generations generating non-agouti mice with a composition of 93.75% C57BL/6; 6.25% 129S4. Similarly, N3 C57BL/6 non-agouti transgenic mice positive for both Tyr-shRNA and CAG-rtTA3 were crossed to yellow-agouti B6.Cg-A^y^/J mice, and resulting *A* ^*y*^ */+* progeny were intercrossed for four generations giving a final composition of 93.75% C57BL/6; 6.25% 129S4. To minimize genetic variability, all comparisons were performed using littermates. Activation of the *Tyr*-shRNA was initiated at the time of conception for all mice, as breeding transgenic males and females were maintained on a 600-mg/kg doxycycline chow (Bio-Serv, Flemington, NJ).

### Genotyping of Mutant Mice

DNA was extracted from tail biopsies taken at P21 using the Quick Genotyping^TM^ DNA Preparation Kit (Bioland Scientific, LLC, Paramount, CA) according to the manufacturer’s instructions. Genomic DNA was used to genotype the mice for germline transmission of (i) the *Tyr* shRNA or the *Luc* shRNA and (ii) the specific rtTA transactivator (R26-rtTA2 or CAG-rtTA3). The mice were also genotyped at the *Col1a1* locus. A 2x Taq PCR Premix (Bioland Scientific) was used for all genotyping reactions. All PCR products were separated through a 2% (wt/vol) agarose gel. The expected sizes of the DNA products for each specific genotyping reaction are: *Tyr*-shRNA: 200 bp (with RBG-1 reverse primer); wild-type *Col1a1*: 220 bp; targeted *Col1a1*: 295 bp; ROSA26 wild-type: 500 bp; ROSA26-rtTA2: 300 bp; wild-type rtTA3: 363 bp; mutant rtTA3: 300 bp.

### Quantification of Total Melanin in shaved mouse hair using Soluene-350 and Absorbance at 492 nm

Dorsal hairs of mice at P50 or P100 were shaved and 1 mg was dissolved overnight in 1 mL of hot (65°C) Soluene-350 (PerkinElmer) and 10% water. Triplicate 150 μL aliquots for each mouse hair sample were analyzed for absorbance at 492 nm as previously described to generate an average absorbance [40–43]. The values for experimental mice were normalized to control mice, and percentage of control was calculated. This quantitation method was used only to compare littermates in order to correlate visual appearance of individual mice with melanin accumulation in the hair.

### Quantification of Total Melanin, Eumelanin, and Pheomelanin in shaved mouse hair

Dorsal hairs of mice at P50 or P100 were shaved and 20 mg was homogenized with a Ten Broeck homogenizer at a concentration of 10 mg/mL. Duplicate 100 μL aliquots were solubilized in Soluene-350 (PerkinElmer, Waltham, MA) [44], and subjected to alkaline hydrogen peroxide oxidation [45], and hydroiodic acid hydrolysis [43] as previously described. Statistical inference for differences in mean outcomes between rtTA3/*Tyr*-shRNA and control were tested using a two-sample t-test with no assumption of equal variances between groups. All tests were stratified by mouse color (black vs. agouti). A Wald-based 95% confidence interval and corresponding P-value for a test of the null hypothesis of no difference in true means between the groups was computed for each outcome. To assess sensitivity of outlying observations, Wilcoxon rank sum tests were also conducted to assess any qualitative difference in findings. No differences were found. Bar plots depicting the observed mean and standard deviation of each outcome along with dot plots depicting the raw experimental data for each group were produced. All statistical analyses were carried out using the R statistical software package (Ver. 3.2.1; [46]).

### RNA isolation and quantitative RT-PCR on cultured cells

RNA isolation and quantitative RT-PCR was employed as previously described [36]. Briefly, a Cells-to-Ct kit (Applied Biosystems, Grand Island, NY) was utilized to lyse the infected B16 cells after selection with puromycin. A high-capacity RNA-to-cDNA kit was then employed to generate cDNA (Applied Biosystems). Solaris qPCR gene expression assays for TYR (AX-012555-00-0200) and β-actin (AX-003451-00-0100) were obtained from Thermo Scientific/Dharmacon RNAi technologies and used with TaqMan Gene Expression Master Mix (Applied Biosystems) to complete the PCR. A 7900HT Fast Real-Time PCR system (Applied Biosystems) and SDS 2.4 (Applied Biosystems) were used to determine *C*_t_ values for each sample. Values were normalized to β-actin using the relative quantification mathematical model (Pfaffl) as previously described [33, 36]. A two-tailed Student's *t*-test was employed to determine statistical significance (**p < 0.01).

### Tissue preparation and immunohistochemistry

Whole mouse skin was harvested from either anesthetized or euthanized mice using a four-mm round punch biopsy (Sklar Instruments, Westchester, PA) and formalin-fixed for 24–48 hours using a 10% w/v formalin solution (Fisher Scientific, Pittsburgh, PA). Whole mouse eyes were harvested from euthanized mice and cut in half using a feather blade, followed by fixation in 10% formalin solution for 24–48 hours. Whole skin samples and eyes were then dehydrated and embedded in paraffin. Next, seven-μm thick skin and eye sections were de-waxed and rehydrated through a graded series of alcohol washes to water. After application to a microscope slide, all samples were dried overnight at 37°C. All samples were stained with hematoxylin and eosin to view general structure. For immunohistochemistry, antigen retrieval was carried out by heating slides to 85°C for 15 minutes in 0.01 M citrate buffer (pH 6) on a hot plate. Endogenous peroxidase activity was quenched with Dual Endogeneous Enzyme Block (Dako, Carpinteria, CA). Protein block was carried out using a Protein Block Solution (Dako). Skin and eye samples were then incubated with anti-GFP or anti-Melan-A primary antibody overnight at 4°C at a 1:1000 and a 1:50 dilution, respectively. Subsequently, samples were incubated with biotinylated secondary antibody for two hours at room temperature, followed by exposure to avidin-biotin enzyme complex (Vector Laboratories, Burlingame, CA). Signal was developed using diaminobenzidine (DAB) as the enzyme substrate (Dako) with a hematoxylin counterstain, followed by a final dehydration (50% ethanol for one minute, followed by two washed in 100% ethanol for one minute each) and mounting with Permount (Fisher Chemicals) at room temperature.

### Immunoblotting- cultured cells

Puromycin-selected B16 mouse melanoma cells were subjected to immunoblotting as previously described [36].

### Immunoblotting- whole mouse skin

Whole mouse skin samples were collected from sacrificed mice using a four-mm punch biopsy (Sklar Instruments) and immediately flash frozen in liquid nitrogen. Skin samples were then ground using a mortar and pestle and lysed in RIPA buffer (Santa Cruz Biotechnologies) supplemented with 1x protease and phosphatase inhibitor cocktail (Thermo Scientific). Lysates were clarified by centrifugation (18,000 x *g* for 10 min at 4°C). The relative concentration of protein in each lysate was quantified using a BCA Protein Assay Kit (Thermo Scientific). A total of 20 μg of protein per sample was separated on a 4–10% Bis-Tris gel (Life Technologies, Carlsbad, CA) under reducing conditions and transferred onto a 0.45-μm PVDF membrane (Millipore, Billerica, MA). Membranes were soaked in a blocking buffer solution composed of TBS (Fisher Scientific), 0.1% Tween-20 (Fisher Scientific) and 5% Apex (Genesee Scientific, Carlsbad, CA) non-fat milk powder. To assess immunoreactivity, either Luminata Forte chemiluminescent detection substrate (Millipore) or HyGLO Quick spray (Denville, Holliston, MA) was used. Protein levels were assessed via densitometry analysis using ImageJ (NIH) [47].

### Electron Microscopy

Whole mouse skin (*n* = 2 per genotype) was harvested from either anesthetized or euthanized mice using a four-mm round punch biopsy and fixed in half-strength Karnovsky’s fixative [48] for 24 hours before being transferred to sodium cacodylate buffer, 0.2M, pH 7.4 (Electron Microscopy Sciences, Hatfield, PA). Tissue was then post-fixed with 1% osmium tetroxide containing 1.5% potassium ferrocyanide. After dehydration, tissues were embedded in EPON and sections were obtained using a RMC-MT6000XL ultra-microtome and stained with uranyl acetate and lead citrate. Sections were viewed and selected images were digitally photographed using a JEOL JEM-1230 transmission electron microscope. For DOPA histochemistry and prior to post-fixation, tissues were incubated in a 0.1%solution of L-DOPA twice for 2.5 hours. The tissues were washed and processed as described above. To assess melanosome maturation, the percentage of melanosomes in the four maturation stages was calculated visually in the electron micrographs in the knockdown and control mice using 12 melanocytes from 2 mice per group totaling ~400 melanosomes per mouse. To differentiate between Stage III and Stage IV melanosomes, stage III melanosomes were characterized as having some melanin deposition and observable melanofilaments, while stage IV melanosomes were categorized as having dark melanin and no observable melanofilaments aberrant or not in morphology.

### RNA isolation on whole mouse skin and Nanostring nCounter Analysis

Whole mouse skin (*n* = 3 per genotype) was harvested from either anesthetized or euthanized mice using a four-mm round punch biopsy and immediately stabilized overnight in RNAlater RNA stabilization reagent (Life Technologies) at 4°C. Skin samples were homogenized using the Precellys24 high-throughput tissue homogenizer (Precellys, Bertin Corporation, Rockville, MD) in hard tissue homogenizing reinforced tubes that contain 2.8 mm ceramic beads (Bertin Corporation). After homogenization, RNA was extracted from each sample using the RNeasy Fibrous Tissue Mini Kit according to the manufacturer’s instructions (Qiagen, Valencia, CA). The quantitative analysis of mRNA was carried out using the nCounter Nanostring Technology (NanoString Technologies, Seattle, WA). NanoString’s nCounter technology is based on direct detection of target molecules using color-coded molecular barcodes, providing a digital simultaneous quantification of the number of target molecules. All data were normalized to three housekeeping genes quantified in the same samples. Total mRNA (100 ng in 5μl) was hybridized overnight with nCounter Reporter (20μl) probes in hybridization buffer and in excess of nCounter Capture probes (5μL) at 65°C for 16–20h. The hybridization mixture containing target/probe complexes was allowed to bind to magnetic beads containing complementary sequences on the Capture Probe. After each target found a probe pair, excess probes were washed followed by a sequential binding to sequences on the Reporter Probe. Biotinylated capture probe-bound samples were immobilized and recovered on a streptavidin-coated cartridge. The abundance of specific target molecules was then quantified using the nCounter Digital Analyzer. Individual fluorescent barcodes and target molecules present in each sample were recorded with a CCD camera by performing a high-density scan (1155 fields of view). Images were processed internally into a digital format and were normalized using the NanoString nSolver software analysis tool. Counts were normalized for all target RNAs in all samples based on the positive control RNA to account for differences in hybridization efficiency and post-hybridization processing, including purification and immobilization of complexes. The average was normalized by background counts for each sample obtained from the average of the eight negative control counts. Subsequently, a normalization of mRNA content was performed based on six internal reference housekeeping genes, including *Gapdh*, β-*Actin* and *Hprt* using nSolver Software.

## Results

### Modulation of *Tyr* expression alters the phenotypic appearance of the mouse hair

**Fig 1:**
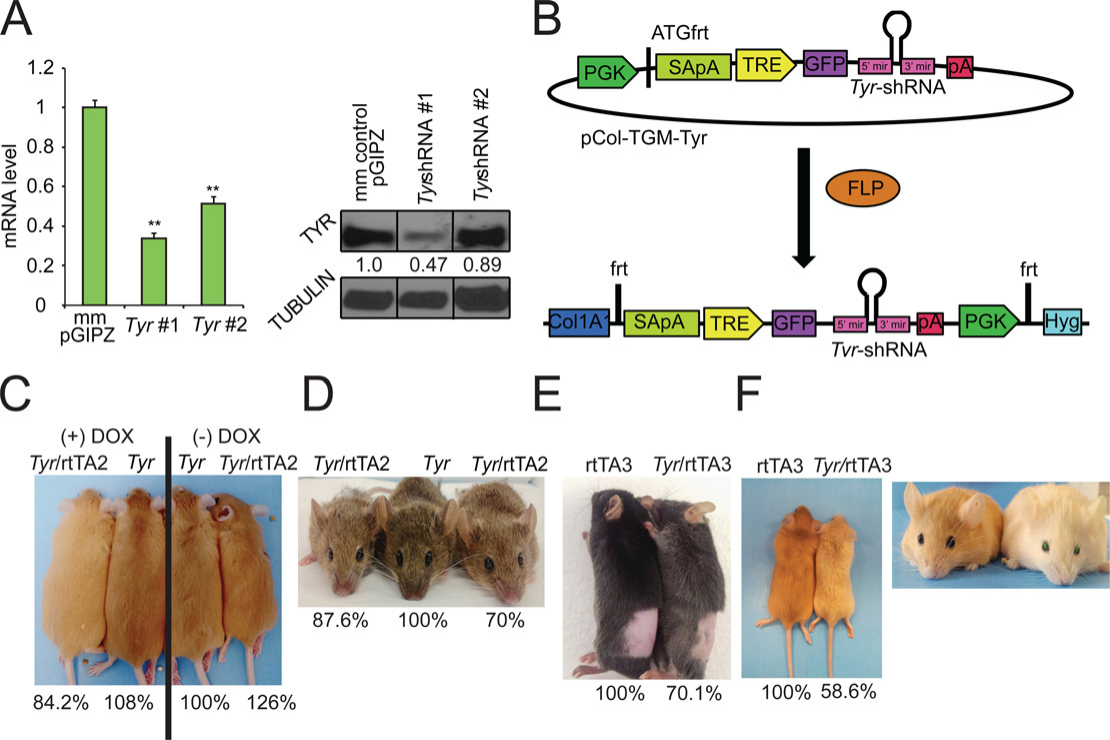
*Tyr* depletion leads to coat color changes in three unique coat color backgrounds. (A) B16 mouse melanoma cells infected with the lentiviral constructs containing individual shRNAs were harvested for quantification of *Tyr* mRNA (left panel) and protein (right panel). The values below the TYR protein bands in the western blot represent the relative intensity of the TYR band normalized to the tubulin band (loading control) for each lane divided by the relative expression of the TYR band in the mismatch-control (mm control) sample. (B) Mouse KH2 ES cells containing a FRT-hygro-pA cassette on chromosome 11 and a reverse tet-transactivator (rtTA) on chromosome 6 were co-electroporated with pCAGGS-FLPe and the targeting vector, pCol-TGM-*Tyr*. Resulting FLPe-mediated recombination between the FRT site at the *Col1a1* locus and the FRT sites present within pCol-TGM-*Tyr* results in colonies that survive hygromycin selection. (C-F) Effect of Dox-mediated Tyr shRNA on coat color was analyzed in (C,F) yellow agouti, (D) white-bellied agouti, or (E) non-agouti (black) mice by comparison of littermates. The genotype of each mouse is listed above each photo. The percentage value below each mouse corresponds to the absorbance at 492 nm for that particular mouse divided by the absorbance at 492 nm for its control littermate, which is set to 100%. (C) Yellow-agouti littermates with the rtTA2 driver; (D) agouti littermates with the rtTA2 driver; (E) black (non-agouti) littermates with the rtTA3 driver and (F) yellow agouti littermates with the rtTA3 driver were shaved on their dorsal side and then photographed. rtTA3 and *Tyr-*shRNA/rtTA3 littermates each maintained on a doxycycline diet were photographed in daylight at P100 to demonstrate the presence of GFP in the eye.

To develop a model to study pigment diversity *in vivo*, we generated a murine model where we could selectively modulate the expression of any gene using a tet-regulatable single-copy shRNA linked to a GFP reporter [38]. First, we utilized an algorithm [35] to identify two shRNA sequences that could effectively silence *Tyr*. Lentiviral constructs that expressed *Tyr* shRNA or *non-targeting* scrambled shRNA (negative control) were used to infect B16 mouse melanoma cells at a MOI of 0.1 to ensure that each cell was only infected with one lentivirus (single copy shRNA) [38]. Quantitative RT-PCR, western blot, and densitometry confirmed that one of the two tested shRNAs (*Tyr-*shRNA #1) efficiently suppressed *Tyr* mRNA (Fig 1A, left panel) and protein expression (Fig 1A, right panel). *Tyr*-shRNA #1 was cloned into the pCol-TGM targeting vector [37] (hereafter referred to as pCol-TGM-*Tyr* shRNA; Fig 1B). To introduce the *Tyr-*shRNA into the mouse genome immediately downstream of the *Col1a1* locus, pCAGGS-Flpe and pCol-TGM-*Tyr* shRNA were co-electroporated into KH2 mouse embryonic stem cells to generate Flpe-mediated recombination between the Frt site at the *Col1a1* locus and the Frt site on the targeting vector (Fig 1B). After recombination, the tetracycline response element (TRE), GFP, and *Tyr*-shRNA are all inserted downstream of the *Col1a1* locus in chromosome 11 in the KH2 cells (Fig 1B), a C57BL/6J x 129S4 F1 (agouti) ES cell line that is pre-engineered to express a reverse tet-trans-activator from the ROSA26 locus on chromosome 6 [37]. Southern blot analysis confirmed the correct integration of the *Tyr*-shRNA into the *Col1a1* locus in 12/12 clones tested (S1A Fig). Modal chromosome number was verified in a sub-set of clones that were then injected into C57BL/6NTac blastocysts, and implanted into CD-1 pseudo-pregnant female foster mice. Chimeric male agouti mice confirmed to contain both the R26-M2rtTA (hereafter referred to as rtTA2) and the *Tyr*-shRNA via genotyping were then bred to eight-week old C57BL/6J females. To verify that the *Tyr*-shRNA was expressed in the hair in a doxycycline-dependent manner, breeding pairs were placed on a 600-mg/kg doxycycline chow prior to conception of litters. Resultant pups were shaved at post-natal day (P) 50 and GFP expression within the hair was monitored by fluorescence microscopy. *Tyr*-shRNA/rtTA2 (*Tyr*-knockdown mice) mice but not *Tyr*-shRNA (control mice) mice exhibited robust expression of GFP in the hair, indicating that *Tyr-*shRNA is expressed within the hair in a tet-regulatable manner (S1B Fig, left two columns).

Having established a system to induce the expression of *Tyr-*shRNA in the hair, we next investigated whether depleting TYR in the hair and skin of *Tyr*-knockdown mice could modulate coat color similarly in mice containing different coat color compositions. To do so, we bred *Tyr*-shRNA;rtTA2 chimeric mice with mice having agouti (*A* ^*w*^) and yellow agouti (*A* ^*y*^) backgrounds as described in methods. We also bred *Tyr*-shRNA mice with mice expressing the CAG-rtTA3 (rtTA3) tet-transactivator protein, which is more effective than the rtTA2 transactivator at driving expression of tet-regulatable elements *in vivo* [49]. The rtTA3 transgene was introduced to non-agouti (black) (*a/a*) and yellow agouti (*A* ^*y*^ */+*) backgrounds as described in methods. Initial studies showed *Tyr*-shRNA/rtTA2 and *Tyr*-shRNA/rtTA3 mice to be fully viable, fertile, and without obvious developmental defects. Pregnant animals and their offspring were fed doxycycline, and progeny were photographed at P50 to make a comparison of coat color in littermates before being shaved and depilated. The shaved hair was dissolved overnight in hot Soluene-350 in 10% water as described in the methods and the absorbance of the dissolved hair was measured at 492 nm, a wavelength at which both eumelanin and pheomelanin absorb [40–44]. These comparisons were done to correlate visual differences observed in individual mice with differences in overall melanin accumulation. We first analyzed the difference between *Tyr*-shRNA/rtTA2 mice maintained on a doxycycline (+ DOX) diet or a non-doxycycline (-DOX) regular diet. Yellow agouti *Tyr*-shRNA/rtTA2 mice on a doxycycline diet consistently demonstrated lighter coat colors compared to *Tyr*-shRNA/rtTA2 littermates fed diet without doxycycline, both visually and spectrophotometrically at P50 (Fig 1C, compare far left mouse with far right mouse, numbers below the pictures correspond only to mice depicted). Similarly, partial depletion of *Tyr* in a white-bellied agouti coat-color background inhibited pigment accumulation in the mouse hair as two littermates with the genotype *Tyr*-shRNA/rtTA2 displayed lighter pigmentation compared to their *Tyr*-shRNA control littermate at P50 (Fig 1D). The agouti *Tyr*-knockdown mice demonstrated 15% and 30% less absorbance at 492 nm compared to the *Tyr*-shRNA (without rtTA2) littermate control, demonstrating that partial *Tyr* depletion moderately inhibits pigment accumulation in the hair (Fig 1D, percentage values below the images correspond only to mice depicted in the images). Non-agouti (black) *Tyr*-shRNA/rtTA3 littermates fed DOX also exhibited significant lightening of coat color after P50 that was dependent on genotype (Fig 1E). Moreover, GFP was easily detected in the hair shaft of *Tyr*-shRNA/rtTA3 black mice, demonstrating the efficiency of the rtTA3 driver (S1B Fig, two right columns). Yellow agouti *Tyr*-shRNA/rtTA3 mice had more pronounced lighter coats and a 40% decrease in pigment accumulation compared with control littermates (Fig 1F, percentages only correspond to littermates shown in the picture). Intriguingly, these mice also developed green eyes (Fig 1F, right panel). Fluorescence microscopy (S1C Fig) and immunohistochemistry (S1D Fig) revealed that this phenotype was related to deposition of GFP within the mouse lens. Importantly, despite variability in the extent of coat lightening in Dox-fed *Tyr*-shRNA; rtTA (2 or 3) mice compared to their littermates, we never observed a lighter coat color in *Tyr*-shRNA single transgenic animals compared with their *Tyr*-shRNA; rtTA double transgenic littermates. Taken together, these results indicate that partial depletion of *Tyr* using inducible shRNAs can consistently modulate pigment accumulation in the hair of mice.

To investigate how the partial depletion of *Tyr* impacts melanin accumulation, we quantified melanin accumulation in hairs of mice containing primarily black coat composition and primarily agouti coat composition using established chemical methods [42, 43, 45, 50]. A comparison of five non-agouti (black) *Tyr*-shRNA/rtTA3 mice with five control mice all maintained on DOX demonstrated that partial depletion of *Tyr* leads to a 10% reduction in eumelanin content (Table 1). Dot plots of the raw experimental data revealed that this observation was not a result of outliers within the dataset (S2 Fig). While *Tyr-*shRNA expression did inhibit eumelanin accumulation in the hair of mice with black coats, the observed differences did not reach statistical significance secondary to the intrinsic variation in eumelanin accumulation observed within experimental mice from different litters. A comparison of 13 agouti *Tyr*-shRNA/rtTA2 mice with 13 agouti control mice, however, yielded no difference in eumelanin accumulation (Table 2), and only an insignificant increase in pheomelanin accumulation (Table 2). A plot of the raw data and secondary sensitivity analyses revealed that these results were not influenced by outliers within the experimental dataset (S3 Fig). It is conceivable that significant differences were not seen in the agouti background because the rtTA2 element is known to be less effective at inducing the expression of shRNA *in vivo* [49] suggesting that *Tyr* knockdown was not as robust as that observed in the rtTA3 mice.

**Table 1:** Knockdown of *Tyr in vivo* is not sufficient to induce significant melanin loss in the black mouse coat.

Outcome	Control (n = 5)	rtTA3/*Tyr*-shRNA (n = 5)	Difference (rtTA3/*Tyr*-shRNA–Cont)		% of Control
	Mean (SD)	Mean (SD)	Diff in Means (95% CI)	*p*-value	
**A500/mg**	0.76 (0.09)	0.71 (0.08)	-0.06 (-0.18, 0.06)	0.332	
**Total melanin (μg/mg)**	81.08 (10.65)	73 (3.72)	-8.08 (-19.5, 3.34)	0.148	90.1%
**PCTA (ng/mg)**	3234.6 (423.9)	2915.6 (148.9)	-319 (-773.6, 135.6)	0.151	
**Eumelanin (μg/mg)**	80.88 (10.6)	72.9 (3.71)	-7.98 (-19.34, 3.38)	0.151	90.1%
**4-AHP (ng/mg)**	23.44 (7.4)	14.04 (6.88)	-9.4 (-19.63, 0.83)	0.071	
**Pheomelanin (μg/mg)**	0.22 (0.084)	0.14 (0.055)	-0.08 (-0.18, 0.02)	0.111	63.6%
**Average Phe/Eu Ratio**	0.003 (0.001)	0.002 (0.001)	0.001 (-0.001, 0.001)	0.185	

Dorsal hairs of non-agouti double transgenic and control mice on continuous doxycycline treatment were shaved and processed for chemical analysis of eumelanin and of pheomelanin based upon their specific degradation products, pyrrole-2,3,5-tricarboxylic acid (PCTA) and 4-amino-3-hydroxyphenylalanine (4-AHP), respectively. Approximately 20 mg of hair was homogenized with a Ten Broeck homogenizer at a concentration of 10 mg/mL and 100 μL aliquots were subjected to Soluene-350 solubilization to quantify total melanin, alkaline hydrogen peroxide oxidation to quantify total PCTA, and hydroiodic acid hydrolysis to quantify 4-AHP in duplicate. The data corresponds to the averages of five control mice (*Tyr*-shRNA only or rtTA driver only) and five *Tyr*-knockdown mice, and the value is the mean of duplicate measurements. Each hair sample was derived from a single mouse. Within group summary statistics represent the arithmetic mean and (standard deviation). Differences in means between rtTA3/*Tyr*-shRNA and control were tested using a two-sample t-test with no assumption of equal variances between groups. P-values correspond to a test of the null hypothesis of no difference in true means between the groups. Percent of control is presented for descriptive purposes and represents the relative mean for rtTA3/*Tyr*-shRNA when compared to control. See also S2 Fig.

**Table 2:** Knockdown of *Tyr in vivo* is not sufficient to induce significant melanin loss in the agouti mouse coat.

Outcome	Control (n = 13)	rtTA2/*Tyr*-shRNA (n = 13)	Difference (rtTA2/*Tyr*-shRNA–Cont)		% of Control
	Mean (SD)	Mean (SD)	Diff in Means (95% CI)	*p*-value	
**A500/mg**	0.69 (0.1)	0.66 (0.15)	-0.03 (-0.14, 0.08)	0.582	
**Total melanin (μg/mg)**	75.92 (11.67)	74.95 (16.44)	-0.97 (-12.49, 10.55)	0.864	98.7%
**PCTA (ng/mg)**	2740.4 (410.0)	2651.4 (615.9)	-89 (-511.6, 333.6)	0.668	
**Eumelanin (μg/mg)**	68.52 (10.26)	66.29 (15.41)	-2.22 (-12.79, 8.35)	0.669	96.8%
**4-AHP (ng/mg)**	824.4 (251.7)	963.1 (215.4)	138.7 (-50.5, 327.9)	0.144	
**Pheomelanin (μg/mg)**	7.41 (2.263)	8.66 (1.95)	1.25 (-0.46, 2.96)	0.143	116.9%
**Average Phe/Eu Ratio**	0.108 (0.025)	0.135 (0.034)	0.027 (0.01, 0.05)	0.027	

Dorsal hairs of agouti mice on continuous doxycycline treatment were shaved and processed for chemical analysis of eumelanin and of pheomelanin based upon their specific degradation products, pyrrole-2,3,5-tricarboxylic acid (PCTA) and 4-amino-3-hydroxyphenylalanine (4-AHP), respectively as described in Table 1. 13 control (*Tyr*-shRNA only or rtTA driver only) and 13 *Tyr*-knockdown mice were analyzed. Within group summary statistics represent the arithmetic mean and (standard deviation). Differences in means between rtTA3/*Tyr*-shRNA and control were tested using a two-sample t-test with no assumption of equal variances between groups. P-values correspond to a test of the null hypothesis of no difference in true means between the groups. Percent of control is presented for descriptive purposes and represents the relative mean for rtTA3/*Tyr*-shRNA when compared to control. See also S3 Fig.

### Disruption of *Tyr* inhibits melanosome maturation

**Fig 2:**
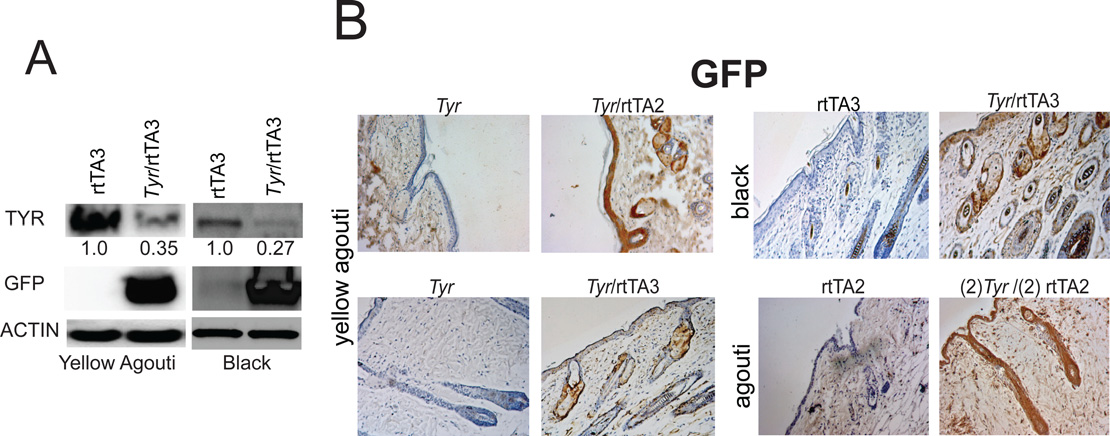
Depletion of TYR protein in skin in *Tyr*-shRNA; rtTA3 mice. (A) Four-mm skin punch biopsies were taken at P100 from three different coat color backgrounds. Total protein was extracted and used to immunoblot for TYR and GFP. The numerical values present below the TYR protein bands in the western blot represent the relative intensity of the TYR band normalized to the beta-actin band (loading control) for each lane divided by the relative expression of the TYR band in the rtTA3 control sample. (B) Four-mm skin punch biopsies taken from the indicated mice at P100 were formalin fixed, dehydrated, and paraffin embedded. Next, seven-μm thick sections of the skin were cut and immunostained for GFP.

To better assess how effectively our *Tyr*-shRNAs could inhibit TYR expression *in vivo*, we performed a more detailed analysis of the expression of the *Tyr*-shRNA and TYR in *Tyr*-shRNA/rtTA2 and *Tyr*-shRNA/rtTA3 knockdown mice. To do so, we performed western blot analysis on skin harvested at P50 from *Tyr*-shRNA/rtTA3 knockdown mice and *Tyr*-shRNA control mice. Western blot analysis indicated knockdown of TYR with simultaneous substantial expression of GFP, providing evidence of *Tyr* knockdown in the skin tissue (Fig 2A). Densitometry analysis showed a 65%-73% reduction in TYR protein (Fig 2A, numerical values under TYR protein bands). We also utilized harvested skin from mice at P50 and performed immunohistochemistry using an anti-GFP antibody. When compared to rtTA2, rtTA3, or *Tyr*-shRNA only littermate controls, *Tyr*-shRNA/rtTA2 and *Tyr*-shRNA/rtTA3 mice showed robust staining of GFP in the epidermis and the hair follicle in all coat color backgrounds, confirming co-expression of *Tyr*-shRNA when mice are fed a DOX diet (Fig 2B). Further IHC experiments demonstrated that GFP expression co-localized with Melan-A expression, indicating that GFP is expressed in melanocytes (S4 Fig).

**Fig 3:**
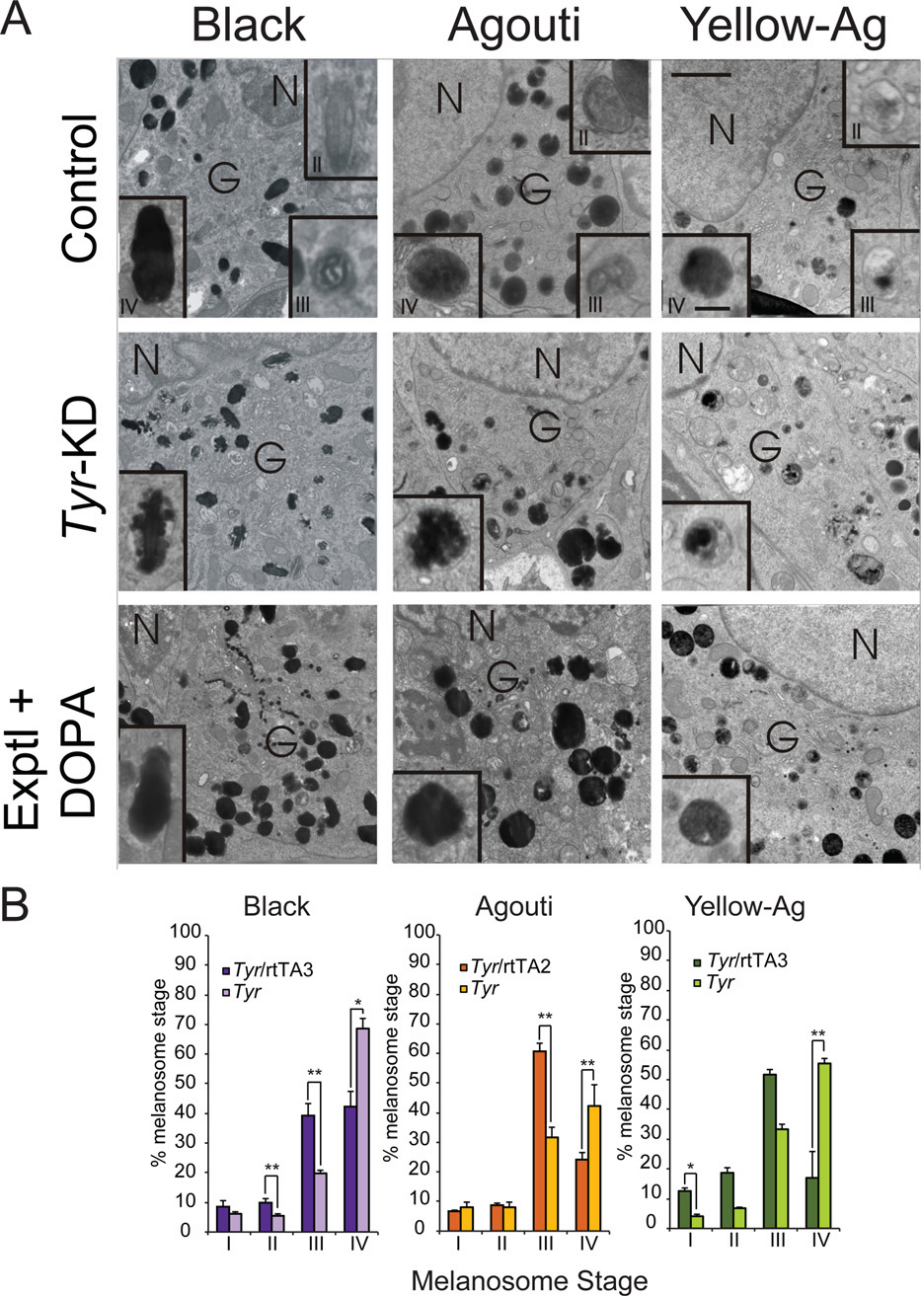
*Tyr* depletion inhibits the normal deposition of melanin within the melanosome. (A) Fresh whole mouse skin was excised from the indicated mice using a four-mm round punch biopsy and fixed in Karnovsky’s fixative before electron microscopy analysis. Melanosomes within the Golgi area of the cell body were evaluated for maturation stage and ultra-structural morphology. DOPA histochemistry was performed to assess the relative activity of tyrosinase within the melanosome. The top row contains the relevant control images for the images listed in the middle and bottom rows, respectively. Images are representative of 15 melanocytes from 2 mice per group. (B) The relative accumulation of stage I-IV melanosomes in each coat color background was quantified as described in the methods. For black and yellow agouti coat colors, the rtTA3 driver was used. For white-bellied agouti coat color, the rtTA2 driver was used. N = nucleus; G = Golgi area. Bar = 0.5 microns (Bar on inset = 1.5 microns). Graph: * = *p* ≤0.05, or ** = *p* ≤0.005, Bars = standard deviation (*n* = 15 melanocytes from 2 mice per group).

Having determined how efficiently the *Tyr*-shRNA could inhibit TYR expression, we next sought to examine whether loss of endogenous *Tyr* affects the structure of the melanosome. Fresh skin harvested from *Tyr*-knockdown mice and appropriate littermate controls on both the white-bellied agouti background and the yellow agouti background were analyzed by transmission electron microscopy (TEM, Fig 3). The respective littermate controls had normal early and late-stage melanosomes that were round with smooth, fibrillar deposits of melanin, consistent with the normal melanosome maturation pattern observed in mice (Fig 3A, top row, center & right) [51]. In contrast, the majority of melanosomes originating from *Tyr*-knockdown mouse skin accumulated melanosome structures characterized by an irregular pattern of melanin deposition, similar to the stage II and III melanosomes of MNT-1 melanoma cells [24]) but different from the stage II and III melanosomes observed in mouse skin (Fig 3A, middle row, center and right images) [51]. Finally, depletion of *Tyr* quantitatively inhibited the maturation of the melanosome, as more stage III, but less stage IV melanosomes were present in *Tyr*-knockdown mice on both agouti coat color backgrounds (Fig 3B, center & right graphs).

To determine if irregular melanin deposition occurs in black mice when *Tyr* is reduced, we harvested fresh skin from *Tyr*-shRNA/rtTA3 mice and control littermates on the black coat background. As before, the control mice displayed normal deposition of melanin within oval melanosomes (Fig 3A, top row, left image and inset). However, the *Tyr*-knockdown mice displayed abnormal melanin deposition that is more reminiscent of melanosome structures observed in MNT-1 melanoma cells as opposed to mouse melanocytes (Fig 3A, middle row, inset of left image) [24, 51]. As with *Tyr*- knockdown mice on the white-bellied agouti and yellow agouti background, we observed that black *Tyr*-knockdown mice possessed significantly fewer stage IV melanosomes compared to littermate controls and significantly more stage III melanosomes, suggesting that TYR is required for complete melanin deposition within the melanosome as well as maturation of the melanosome (Fig 3B, left graph). These ultra-structural observations suggest that knockdown of endogenous *Tyr* may have a more drastic effect on the morphology of the melanosome in agouti and yellow agouti mice then in black mice (Fig 3A, compare inset of middle image in left column to insets of middle images in center and right columns). However, upon addition of DOPA, the irregular melanin deposition in the *Tyr-*knockdown mice in all three genotypes was not apparent. Instead, the melanosomes resembled the melanosomes of the respective control littermates (Fig 3A, compare top row images to bottom row images). These results indicate that the block in melanosome maturation could be overcome *in vitro* by adding unlimited substrate. To verify that the differences observed were not secondary to differences in melanosome density between *Tyr*-knockdown mice and control mice, we quantified and averaged the number of melanosomes per one hundred microns^2^ in fifteen melanocytes from two mice per group. We found no significant differences in the melanosome density between *Tyr*-knockdown mice and controls in all three coat colors, demonstrating that while the partial depletion of *Tyr* is sufficient to decrease total amount of stage IV melanosomes, it is not sufficient to alter the total number of melanosomes (S1 Table).

While melanosome maturation occurs predominantly in the Golgi area, mature melanosomes are localized to the dendritic processes [16, 23]. As the observed phenotype in the *Tyr*-knockdown mice was restricted to terminal melanosomes, we examined the morphology of melanosomes in the dendritic tips of melanocytes. While the melanosomes in the dendrites of the control mice displayed a normal morphology (S5A Fig, upper row), melanosomes in the dendrites of *Tyr*-knockdown mice of all three coat colors displayed the same ruffled morphology as was visualized in the Golgi area (S5A Fig, lower row).

### The observed phenotypes are reversible and not a consequence of shRNA expression

**Fig 4:**
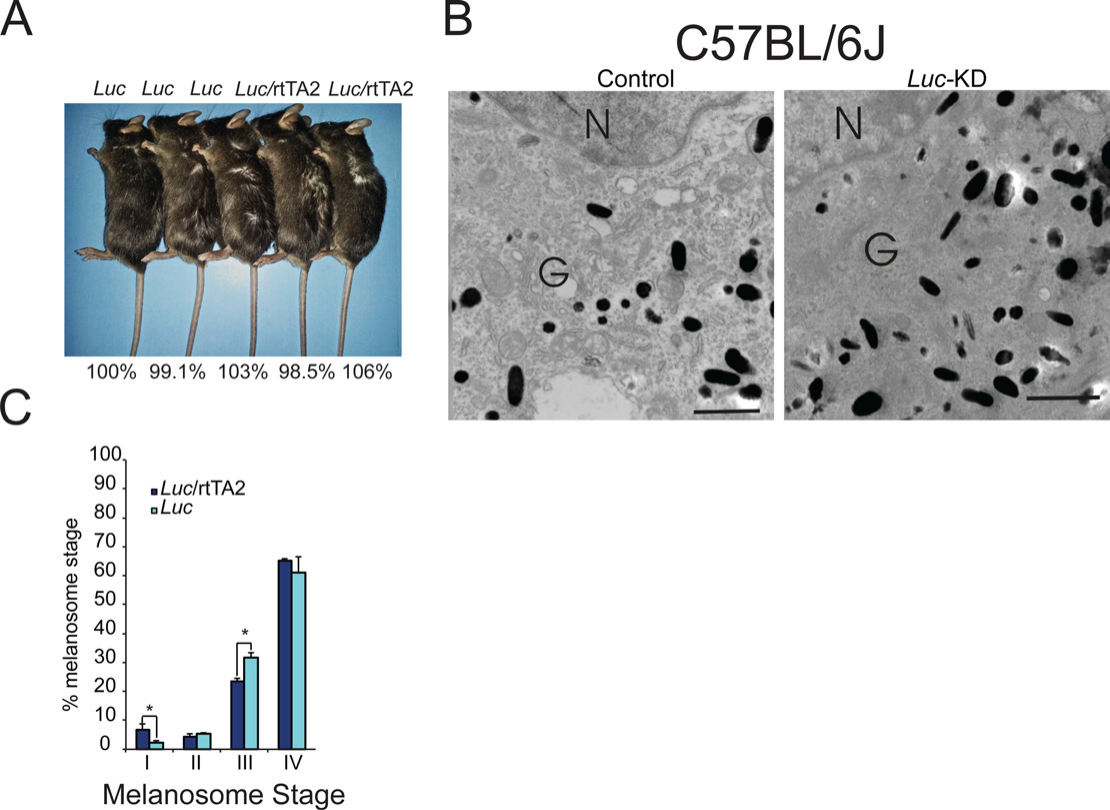
Expression of a control shRNA does not affect pigment accumulation or melanosome maturation. (A) Coat color in *Luc*-knockdown mice and appropriate control mice was compared both visually (genotypes of each mouse are listed above each photo) and spectrophotometrically (the percentage value below each mouse corresponds to the absorbance at 492 nm for that particular mouse divided by the absorbance at 492 nm for its control littermate, which is set to 100%). (B) Fresh whole mouse skin was excised from *Luc*-knockdown mice and corresponding control mice using a four-mm round punch biopsy and fixed in Karnovsky’s fixative before electron microscopy analysis. Melanosomes within the Golgi area of the cell body were evaluated for maturation stage and ultra-structural morphology. Images are representative of 15 melanocytes from 2 mice per group. (C) The melanosomes were also quantified as described in Fig 3B. N = nucleus; G = Golgi area. Bar = 0.5 microns (Bar on inset = 1.5 microns). Graph: * = *p* ≤0.05. Bars = standard deviation (*n* = 2 mice per group, 15 melanocytes per mouse analyzed).

To verify that the phenotypes observed in our *Tyr*-knockdown mice were not due to possible side effects caused by GFP expression, we generated mice on the black background that expressed an shRNA directed towards *Luciferase* (*Luc*), a gene that is not expressed in the mouse. First, we examined *Luc*-knockdown mice and appropriate control mice and determined that there were no visual differences in coat color between the two groups (Fig 4A). Next, we analyzed shaved hairs from *Luc*-knockdown mice and control mice using the previously described melanin assay and found no differences in absorbance at 492 nm, indicating that expression of GFP does not affect the production of melanin within the mouse hair follicle (Fig 4A, percentage values below image). To determine whether the expression of a control shRNA or GFP affected the deposition of melanin within the melanosome, fresh skin harvested from *Luc* shRNA/rtTA2 mice and appropriate *Luc-*shRNA controls on the black background and was analyzed by TEM. Melanosomes from *Luc*-shRNA/rtTA2 mice demonstrated no differences in morphology when compared to melanosomes obtained from *Luc-*shRNA only mice (Fig 4B). Both the *Luc*-shRNA and the *Luc*-shRNA/rtTA2 mice contained appropriate numbers of both early and late stage melanosomes, with stage IV melanosomes comprising the majority of the total melanosome number (Fig 4C).

### The partial depletion of *Tyr in vivo* affects the expression of key genes involved in melanogenesis

**Fig 5:**
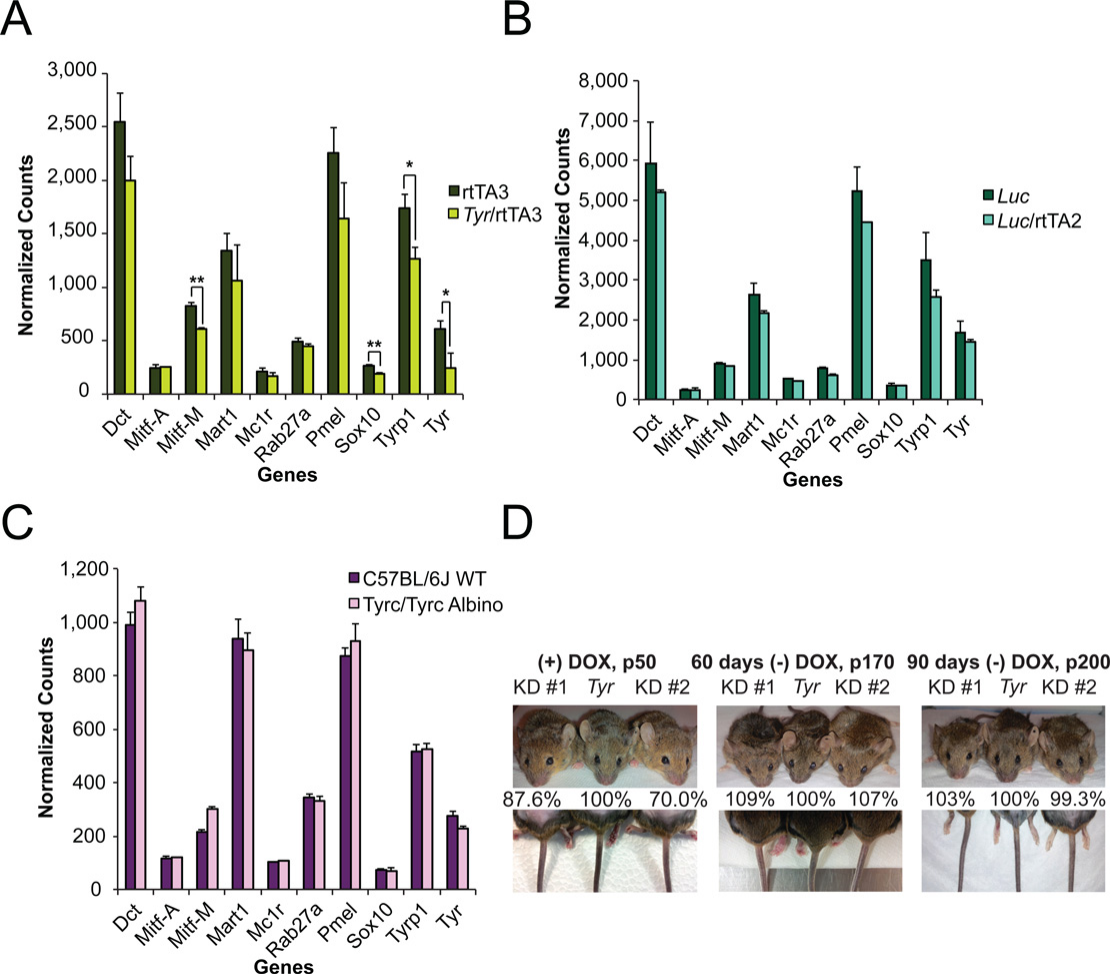
The partial depletion of *Tyr* impacts key genes involved in melanogenesis. Four-mm skin punch biopsies were acquired from anesthetized (A) control rtTA3 mice and experimental *Tyr*-shRNA/rtTA3 mice, (B) control *Luc*-shRNA mice and experimental *Luc*-shRNA/rtTA2 mice, and (C) control C57BL/6J wild-type and *Tyr* ^*c*^ */Tyr* ^*c*^ albino mice and immediately stabilized in RNAlater. Extracted RNA was subjected to Nanostring analysis on the genes listed below the bar graphs. Data shown for all three Nanostring bar graphs (A-C) is mean normalized counts of mRNA for each gene (*n* = 3 mice as indicated by the error bars). * = *p* ≤0.05 or ** = *p* ≤ 0.01. (D) Three littermates (genotypes from left to right as shown in graphs above each figure) on continuous DOX feed were photographed at P50 (left images). After P110, DOX was removed from the diet for 60 days and the mice were photographed once more (middle images). Finally, the mice were fed a DOX-free diet for an additional 30 days and a final set of photographs were taken (right images). Pigment accumulation was measured spectrophotometrically as described in the methods. The percentage value below each mouse corresponds to the absorbance at 492 nm for that particular mouse divided by the absorbance at 492 nm for its control littermate, which is set to 100%.

To assess whether the partial depletion of *Tyr* affected the expression of genes that play key roles in melanogenesis, we harvested fresh whole mouse skin using a four-mm round punch biopsy and subjected the extracted RNA to Nanostring nCounter analysis. Interestingly, knockdown of *Tyr* resulted in a slight decrease in the expression levels of *Dct*, *Pmel5*, and *Mart1* compared to control samples (Fig 5A). *Tyr* depletion lead to a significant decrease in the expression levels of *Mitf-M*, *Sox10*, *Tyrp1* and, as expected, *Tyr* (Fig 5A). These results indicate that the mechanism by which *Tyr* depletion impacts the deposition of melanin within the melanosome is secondary to an effect of TYR levels on the expression of other melanogenesis genes. Finally, depletion of *Luc* produced no significant change in expression of the same genes analyzed in *Tyr*-KD mice, demonstrating that the changes in expression resultant upon *Tyr* knockdown are specific to *Tyr* depletion (Fig 5B).

To better understand how *Tyr* knockdown affects the expression of *Mitf-M*, *Sox10*, and *Tyrp1*, we compared the expression of these genes and *Tyr* between C57BL/6J wild-type mice and *Tyr* ^*c*^ */Tyr* ^*c*^ mice, which express a mutant form of TYR that is trapped in the ER [52]. Interestingly, *Tyr* ^*c*^ */Tyr* ^*c*^ mutant mice did not have decreased expression of *Tyr*, *Sox10*, *Mitf-M*, or *Tyrp1* (Fig 5C), suggesting that the phenotypes observed in our *Tyr* knockdown mice were a direct consequence of reduced wild-type *Tyr* mRNA or a consequence of decreased levels of TYR in the melanosome. We had previously observed that siRNA-mediated *Tyr* knockdown also inhibited MITF expression, and validated that this phenotype could be recapitulated by three different siRNAs (S6 Fig). Taken together, these results suggest that the effects of *Tyr* knockdown on MITF were not an off-target phenomenon *in vitro*, making it less likely that the phenotypes observed with the *Tyr*-shRNA *in vivo* were an off-target phenomenon.

Finally, to further verify that the observed phenotypes *in vivo* were reversible, we performed a longitudinal study in which we monitored coat color in three littermates during and after the administration of a DOX diet. At P50, the two *Tyr*-knockdown white-bellied agouti mice demonstrated less pigment in their hair both visually (Fig 5D, left upper image) and spectrophotometrically (numerical values below image, 12.4% and 30% less absorbance at 492 nm when compared to the littermate control, respectively). The *Tyr-*knockdown mice also exhibited visually less pigment in their tails when compared to their littermate control while on a DOX diet (Fig 5D, left lower image). At P110, mice were returned to a normal diet without DOX. Sixty days after removing DOX, the coat color of the *Tyr*-knockdown mice began to return to levels comparable to the control littermate (Fig 5D, middle upper image). At this point, we utilized shaved hair samples to establish that both *Tyr*-knockdown mice had no differences in absorbance at 492 nm when compared to the *Tyr*-shRNA alone littermate (Fig 5D, percentage values below image). While there were no differences in hair pigmentation at P110, there was still less pigment in the tails of the *Tyr*-knockdown mice as compared to their littermate controls (Fig 5D, lower middle image). At 90 days post DOX removal, neither *Tyr*-knockdown mouse showed any visible differences in coat or tail pigmentation when compared to the control littermate, demonstrating the reversibility of the tet-inducible shRNA-knockdown mouse model (Fig 5D, upper and lower right images). Similar to our observations at 60 days post DOX removal, absorbance values at 492 nm for both *Tyr*-knockdown mice were at control levels at 90 days post DOX removal (Fig 5D, numerical values below right image). Taken together, these studies indicate that the phenotypes observed in the *Tyr*-knockdown mice are reversible.

## Discussion

This study demonstrates the utility of a conditional and reversible gene expression knockdown system to investigate the partial loss of function of gene products involved in pigmentation in an experimental system. Human skin pigmentation is a highly complex trait that relies upon the expression of numerous gene products and environmental influences to generate the observed phenotype [6, 9]. Despite the complete sequencing of the human genome and recent functional genomic studies that have identified novel genes that affect pigment production *in vivo*, it is still not fully understood how pigment gene diversity leads to skin color diversity. To gain a deeper understanding of how alterations in individual genes contribute to pigment diversity, we employed a novel inducible shRNA mouse model to subtly alter TYR protein levels *in vivo*. Depletion of 65–75% of endogenous TYR (Fig 2A) reduced the amount of eumelanin produced in the black mouse coat albeit not significantly (Table 1 and S2 Fig). Depletion of *Tyr* in agouti mice resulted in a slight increase in pheomelanin with no observable change in eumelanin accumulation (Table 2 and S3 Fig). Pairwise comparisons between control and *Tyr*-knockdown littermates demonstrated visual coat color differences as well as differences in absorbance at 492 nm (Fig 1C–1F), but advanced chemical methods showed no statistically significant difference in total melanin content when all mice from a particular coat color were considered (Tables 1 and 2). The lack of statistical significance is most likely due to the variation in melanin accumulation between mouse litters and the variability of *Tyr* knockdown in the inducible shRNA mouse model. Interestingly, we found that the experimental agouti mice with *Tyr* knockdown demonstrate a slight increase in pheomelanin pigment when compared with their control counterparts. Although this observation requires extensive analysis, it might implicate a role for TYR protein in regulating the eumelanin to pheomelanin switch, as has been suggested by other studies [53, 54]. Other published studies using inbred mouse strains have demonstrated a role for *Tyrp1* in regulating pheomelanin production [40]. While our 2008 study (S6 Fig) did not directly test whether *Tyr* siRNA affected TYRP1 expression in human cells, we did observe that *Tyr* shRNA did inhibit the expression of *Tyrp1* in mouse skin [33]. Future studies will be needed to confirm the relationship between *Tyr* and *Tyrp1* knockdown and the eumelanin to pheomelanin switch.

Extensive studies have characterized mutations at the *Tyr* (albino) locus in mice. Multiple mutations within the gene have demonstrated the functions and interactions of TYR with other proteins [55]. Surprisingly, none of the readily available mutations that have been characterized address how modulation of *Tyr* expression may contribute to melanin deposition within the melanosome. Some mutations within the *Tyr* gene result in an albino phenotype associated with lack of TYR activity, including the *Tyr* ^*c*^ */ Tyr* ^*c*^ and *Tyr* ^*c-2J*^/ *Tyr* ^*c-2J*^ homozygous strains [52]. However, both mutations are considered null as the mutated protein is inactive *in vivo* and is retained within the endoplasmic reticulum, thus prohibiting an understanding of how changing levels of TYR may affect melanin deposition [52]. Similarly, the spontaneous *platinum* mutation in the *Tyr* locus produces mice that are very pale with pink eyes in the C57BL/6 background (*Tyr* ^*c-p*^ */Tyr* ^*c-p*^ *)* [52]. Mice with this mutation possess a TYR protein that is mutated at the carboxyl terminal of the protein [56, 57]. This mutation leads to the presence of a premature stop codon and lack of the essential di-leucine protein sorting motif on the cytoplasmic tail, which causes misrouting of TYR to the cell surface as opposed to the proper melanosomal location [56, 58]. Thus, although the *platinum* mutation leads to decreased melanin in the mouse coat, it does not allow for an analysis of how changing levels of TYR may affect melanin deposition. A third spontaneous mutation in the *Tyr* locus, *Himalayan*, leads to a beige coat color phenotype in conjunction with darker pigmentation on the extremities [59]. Although an excellent model to study thermosensitive protein function, it is not helpful in understanding how TYR contributes to diversity in pigmentation. Finally, the *chinchilla* mouse mutant (*Tyr* ^*c-ch*^ */Tyr* ^*c-ch*^ *)* results from a G-to-A point mutation at nucleotide +1523, which leads to the substitution of alanine for threonine at position +482 [60]. These mice are very phenotypically similar to mice that are wild-type at the *Tyr* locus, possessing black eyes and a very dark gray hair coat [52]. Interestingly, *chinchilla* mice have greatly reduced TYR activity (three-fold) [52] and it has been suggested that *chinchilla* TYR is less stable than that of the wild-type enzyme [61]. TEM studies on *chinchilla* hair bulb melanocytes showed a large number of stage II-III melanosomes without melanin deposition, [62] also implicating a role for TYR in influencing melanosome maturation.

In this study we examine the consequences of partial depletion of wild-type *Tyr* mRNA and protein on melanin accumulation and melanosome maturation. Using our shRNA transgenic system, we partially depleted TYR protein at a level that is sufficient to alter coat color but not sufficient to significantly alter melanin accumulation (Figs 1C–1F and 2A, Tables 1.and 2). Ultrastructural analysis revealed that the melanosomes within these mice did not have a smooth peripheral contour, suggesting that melanin is accumulating around intraluminal vesicles within melanosomes (Fig 3A). TYR has been suggested to localize to intraluminal vesicles within melanosomes [63] and roles for intralumenal vesicles as sites of melanin deposition have been described previously [64, 65]. Taken together, these results support a model in which levels of TYR protein within the stage III melanosome can influence both the pattern and degree of melanin deposition within the fully-pigmented stage IV melanosomes *in vivo*. These observations are distinctly different from the albino mice described in the literature, which did not exhibit a similar phenotype most likely because they had no TYR within the melanosome. Finally, our findings that the partial depletion of TYR affects the successful formation of the end stage melanosome and furthermore the visual coat color in mice also correlate well with previous research showing that the modulation of the pattern of melanin deposition within the melanosome in wild turkeys and violet-backed starlings leads to a diversity in plumage color [66].

To investigate the etiology of this melanosome phenotype further, the gene expression pattern of *Tyr* shRNA transgenic mice, *Luc* shRNA transgenic mice, *Tyr* ^*c*^ */ Tyr* ^*c*^ mice, and C57BL/6J mice were compared. Gene expression analysis determined that *Tyr* depletion significantly modulated the expression of *Mitf-M*, *Sox10*, and *Tyrp1* (Fig 5A) while *Luc* shRNA had no effect on the expression of *Mitf-M*, *Sox10*, and *Tyrp1* (Fig 5B). These results indicate that the effects of the shRNA on *Mitf-M*, *Sox10*, *and Tyrp1* were not a consequence of the overexpression of shRNA or GFP in the hair of the *Tyr*-knockdown mice. To determine whether the effects of the *Tyr*-shRNA on these genes were a consequence of decreased *Tyr* expression or decreased TYR activity in the melanosome, we compared the expression of these genes between isogenic *Tyr* ^*c*^ */ Tyr* ^*c*^ mice and C57BL/6 mice (Fig 5C). Interestingly, we observed that there was no change in *Mitf-M*, *Sox10*, nor *Tyrp1* between these mice. Previously published work demonstrated that *Tyr* depletion in MNT-1 cells using multiple different siRNAs (S6 Fig) also inhibited *MITF-M* expression [33]. While it is still conceivable that the observed phenotype in our *Tyr* knockdown mice is an unpredicted *in vivo* specific off target effect, the gene expression relationship uncovered by our studies is more likely a consequence of the decreased level of *Tyr* mRNA/TYR protein within the melanosome of these mice. Other published studies have demonstrated that overexpression of *Tyr* in mouse fibroblasts is sufficient to induce the formation of melanin and what may be melanosome precursors or melanosome-like organelles [67], identifying a role for *Tyr* expression in driving the expression of other melanosome genes. Taken together, these results are consistent with a potential role for either *Tyr* mRNA levels or the level of TYR within the melanosome as modulators of the expression of other melanosome components, specifically *MITF-M*, which regulates the expression of many genes that modulate melanosome structure [68–71]. These observations are consistent with other studies that have also suggested that inhibiting melanosome maturation can feedback to regulate the expression of genes involved in melanogenesis [72, 73].

The inducible and reversible shRNA mediated knockdown transgenic mouse model has several advantages over existing experimental systems. First, the model can be generated rapidly by directed recombination once a suitable shRNA sequence targeting the relevant pigment gene is identified [34]. Second, when the shRNA containing mice are crossed with mice expressing the CAG-rtTA3 driver, the double-transgenic progeny can express high levels of the shRNA (based on GFP expression) in the hair follicle and epidermis under the tight control of a doxycycline-inducible promoter (Fig 2B). Intriguingly, the CAG-rtTA3 driver is not only efficient at driving shRNA expression within the epidermis but can also drive shRNA expression in the mouse lens (S1C and S1D Fig), enabling investigators to study the function of proteins within the lens. Most importantly, this model generates partial loss-of-function phenotypes in contrast to the complete loss of function phenotypes observed in knockout mice. This unique feature allows investigators to quantify the impact of depletion of mRNA and protein on melanogenesis that normally could not be studied in null or conditional mutant null models because the knockout is lethal. On the other hand, this feature also makes these mice slightly more troublesome to characterize, as the level of shRNA expression and knockdown can vary between experimental animals. Despite these limitations, we illustrate the power of this model by using it to demonstrate that TYR controls the pattern of melanin deposition within the melanosome. Future studies will utilize this system to better quantify the relative contributions of individual genes to pigment diversity *in vivo*.

## Supporting Information

S1 Fig (A) Total genomic DNA was extracted from the parental KH2 cell line ("KH2"), or from hygromycin-resistant KH2 clones (1–12) electroporated with a shRNA targeting *Tyr*. DNA's were digested with SpeI and subjected to Southern analysis using a 0.85kb probe from the *Col1a1* locus. The sizes of the DNA fragments detected with this genomic DNA probe are wild-type allele (6.2kb); Frt-Pgk-neo allele (6.7kb); and Flp-in (*Tyr*-shRNA containing) allele (4.1kb). (B) To visualize GFP expression in the hair shaft, shaved dorsal hairs taken at P50 from an agouti *Tyr*-shRNA/rtTA2 (*Tyr/rtTA2*) and a *Tyr*-shRNA (*Tyr*) control littermate (left two columns) and a non-agouti (black) *Tyr*-shRNA/rtTA3 (*Tyr/rtTA3*) and a *Tyr*-shRNA (*Tyr*) control littermate (right two columns) were affixed to slides and subjected to both bright field microscopy and fluorescence microscopy with identical exposure times. (C) Brightfield microscopy was used to photograph the eyes of littermates to demonstrate the presence of GFP in the absence of fluorescence (left column images, middle column images). To verify that the green color in the eye was derived from GFP, eyes were also visualized using fluorescence microscopy (right column images). (D) Four-mm skin punch biopsies taken from the indicated mice at P100 were formalin fixed, dehydrated, and paraffin embedded. Seven μm thick sections of the skin were cut and stained for hematoxylin and eosin (top row), immunostained without primary GFP antibody present (second row down), or immunostained with primary GFP antibody (third row down) to demonstrate the GFP expression throughout the eye of *Tyr*-knockdown mice (right column). The fixed and dehydrated eyes were also subjected to Fontana Masson staining (F-M) to visualize melanin levels (bottom row). Ln = lens, rpe = retinal pigment epithelium. (TIF)

S2 Fig Bar and dot plots for black mice. Bar plots depict the arithmetic mean and (standard deviation) for the rtTA3/*Tyr*-shRNA and control groups (actual numeric values given at the top of each subfigure). Dorsal hairs of C57BL/6J mice on continuous doxycycline treatment were shaved and processed for chemical analysis of eumelanin and of pheomelanin based upon their specific degradation products, pyrrole-2,3,5-tricarboxylic acid (PCTA) and 4-amino-3-hydroxyphenylalanine (4-AHP), respectively. Approximately 20 mg of hair was homogenized with a Tenbroeck homogenizer at a concentration of 10 mg/mL and 100 μL aliquots were subjected to Soluene-350 solubilization to quantify total melanin, alkaline hydrogen peroxide oxidation to quantify total PCTA, and hydroiodic acid hydrolysis to quantify 4-AHP in duplicate. The data corresponds to the averages of five control mice (*Tyr*-shRNA only or rtTA driver only) and five *Tyr*-knockdown mice on the C57BL/6J background, and the value is the mean of duplicate measurements. Each hair sample was derived from a single mouse. ‘X’ symbols on the left of each bar represent the actual data observed for each group.
(TIF)

S3 Fig Bar and dot plots for agouti mice. Bar plots depict the arithmetic mean and (standard deviation) for the rtTA3/*Tyr*-shRNA and control groups (actual numeric values given at the top of each subfigure). Dorsal hairs of agouti mice on continuous doxycycline treatment were shaved and processed for chemical analysis of eumelanin and of pheomelanin based upon their specific degradation products, pyrrole-2,3,5-tricarboxylic acid (PCTA) and 4-amino-3-hydroxyphenylalanine (4-AHP), respectively as described in Table 1. 13 control (*Tyr*-shRNA only or rtTA driver only) and 13 *Tyr*-knockdown mice were analyzed. ‘X’ symbols on the left of each bar represent the actual data observed for each group.
(TIF)

S4 Fig GFP co-localizes with Melan-A in the mouse skin. Four-mm skin punch biopsies taken from the indicated mice at P50 were formalin fixed, dehydrated, and paraffin embedded. Seven-μm thick sections of the skin were cut and stained for hematoxylin and eosin (top row), immunostained for GFP (middle row) and immunostained for Melan-A (bottom row).
(TIF)

S5 Fig *Tyr*-knockdown mice also display aberrant melanosome structure within the melanocyte dendrites in all three mouse coat colors. Fresh whole mouse skin was excised from *Tyr*-knockdown mice and corresponding control mice using a four-mm round punch biopsy and fixed in Karnovsky’s fixative before transmission electron microscopy analysis. Melanosomes within the dendrites of the melanocyte were then evaluated for ultrastructural morphology. Images are representative of 15 melanocytes from 2 mice per group. Bar = 2.5 microns.
(TIF)

S6 Fig Multiple independent siRNAs against *TYR* impact MITF protein accumulation. The impact of multiple independent siRNAs (1, 2, 3) targeting *TYR*, on TYR and MITF protein accumulation was assessed by immunoblot and compared to a control, mismatch siRNA (C). The impact of the given siRNAs on the protein accumulation of TYR and MITF was quantitated by densitometry (numbers below the corresponding blots).
(TIF)

S1 Table The partial depletion of *Tyr in vivo* does not affect the melanosome density within the mouse melanocyte. Fresh whole mouse skin was excised from the indicated mice using a four-mm round punch biopsy and fixed in Karnovsky’s fixative before electron microscopy analysis. The number of melanosomes per area for 15 melanocytes from 2 mice per mouse coat color and genotype were determined.
(DOC)

S2 Table List of Antibodies Used in Experiments. *IHC-P =* immunohistochemistry, paraffin-embedded tissue. *WB =* Western blot
(DOC)

S3 Table List of primers used for genotyping and their sequences, 5’ to 3’. (DOC)

S4 Table Genotype of loci with major effect on coat color in ES cells and mice used in this study. A—agouti, chromosome 2. B—tyrosinase related protein 1 (*Tyrp1*), chromosome 4. C—Tyrosinase (*Tyr*), chromosome 7. p—oculocutaneous albinism II (Oca2), aka pink-eyed dilution, chromosome 7.
(DOCX)
