# Supplementary figures and images for: Tyrosinase Depletion Prevents the Maturation of Melanosomes in the Mouse Hair Follicle
Source: PLoS One. 2015 Nov 30;10(11):e0143702. doi: 10.1371/journal.pone.0143702 (PMC4664286; doi:10.1371/journal.pone.0143702)

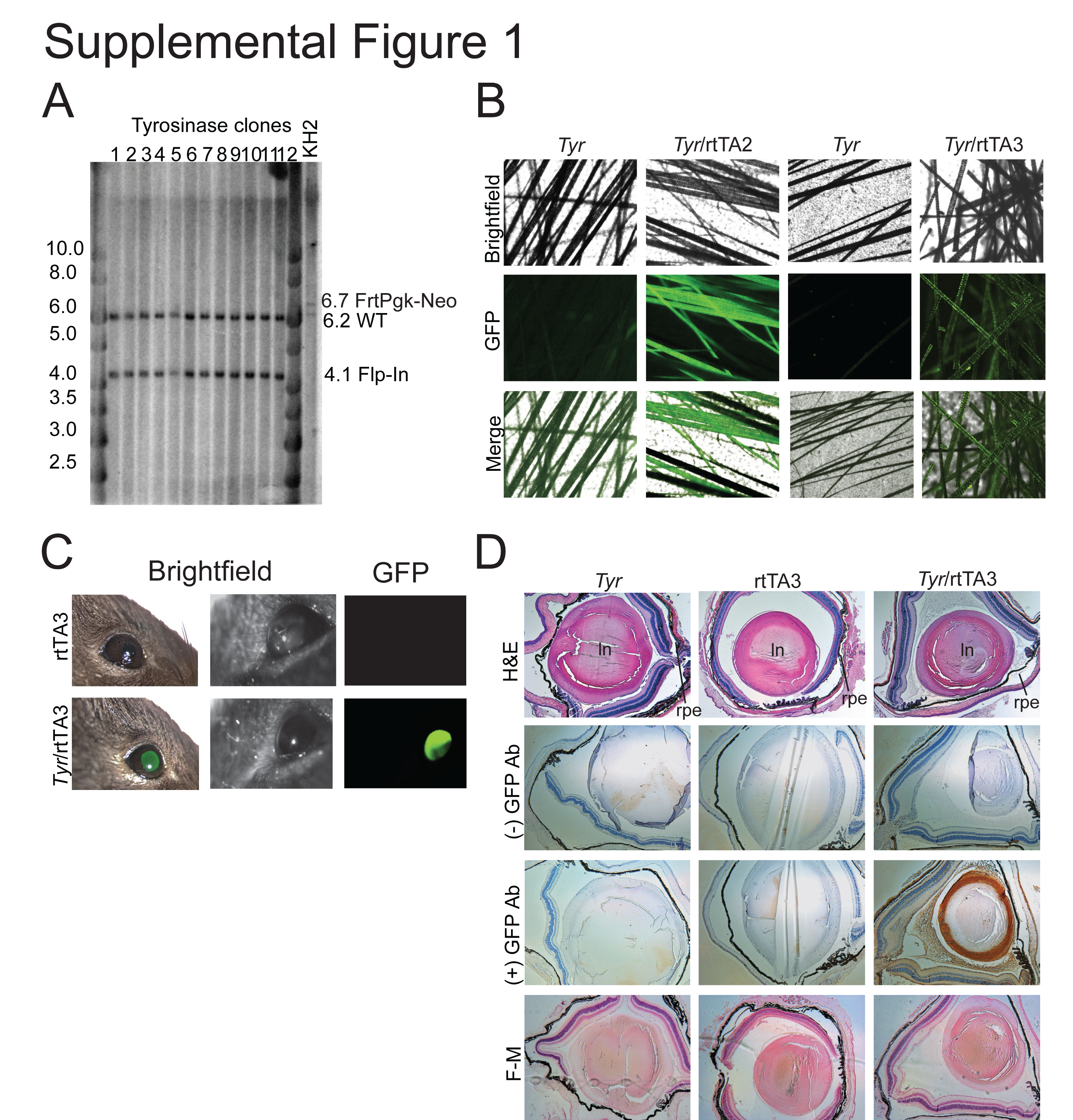

Supplement: S1 Fig — (TIF) [file pone.0143702.s001.tif]

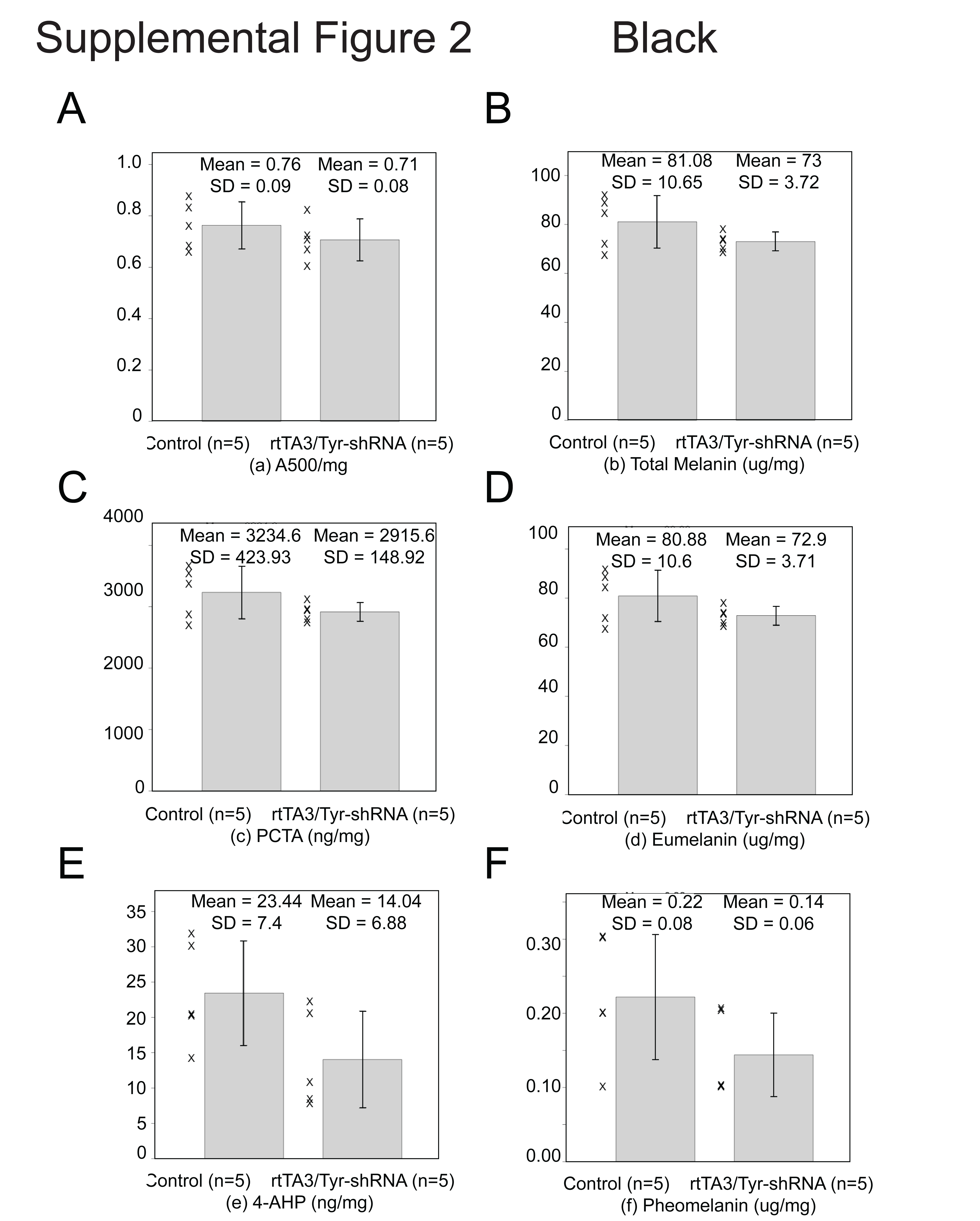

Supplement: S2 Fig — Bar plots depict the arithmetic mean and (standard deviation) for the rtTA3/Tyr-shRNA and control groups (actual numeric values given at the top of each subfigure). Dorsal hairs of C57BL/6J mice on continuous doxycycline treatment were shaved and processed for chemical analysis of eumelanin and of pheomelanin based upon their specific degradation products, pyrrole-2,3,5-tricarboxylic acid (PCTA) and 4-amino-3-hydroxyphenylalanine (4-AHP), respectively. Approximately 20 mg of hair was homogenized with a Tenbroeck homogenizer at a concentration of 10 mg/mL and 100 μL aliquots were subjected to Soluene-350 solubilization to quantify total melanin, alkaline hydrogen peroxide oxidation to quantify total PCTA, and hydroiodic acid hydrolysis to quantify 4-AHP in duplicate. The data corresponds to the averages of five control mice (Tyr-shRNA only or rtTA driver only) and five Tyr-knockdown mice on the C57BL/6J background, and the value is the mean of duplicate measurements. Each hair sample was derived from a single mouse. ‘X’ symbols on the left of each bar represent the actual data observed for each group. (TIF) [file pone.0143702.s002.tif]

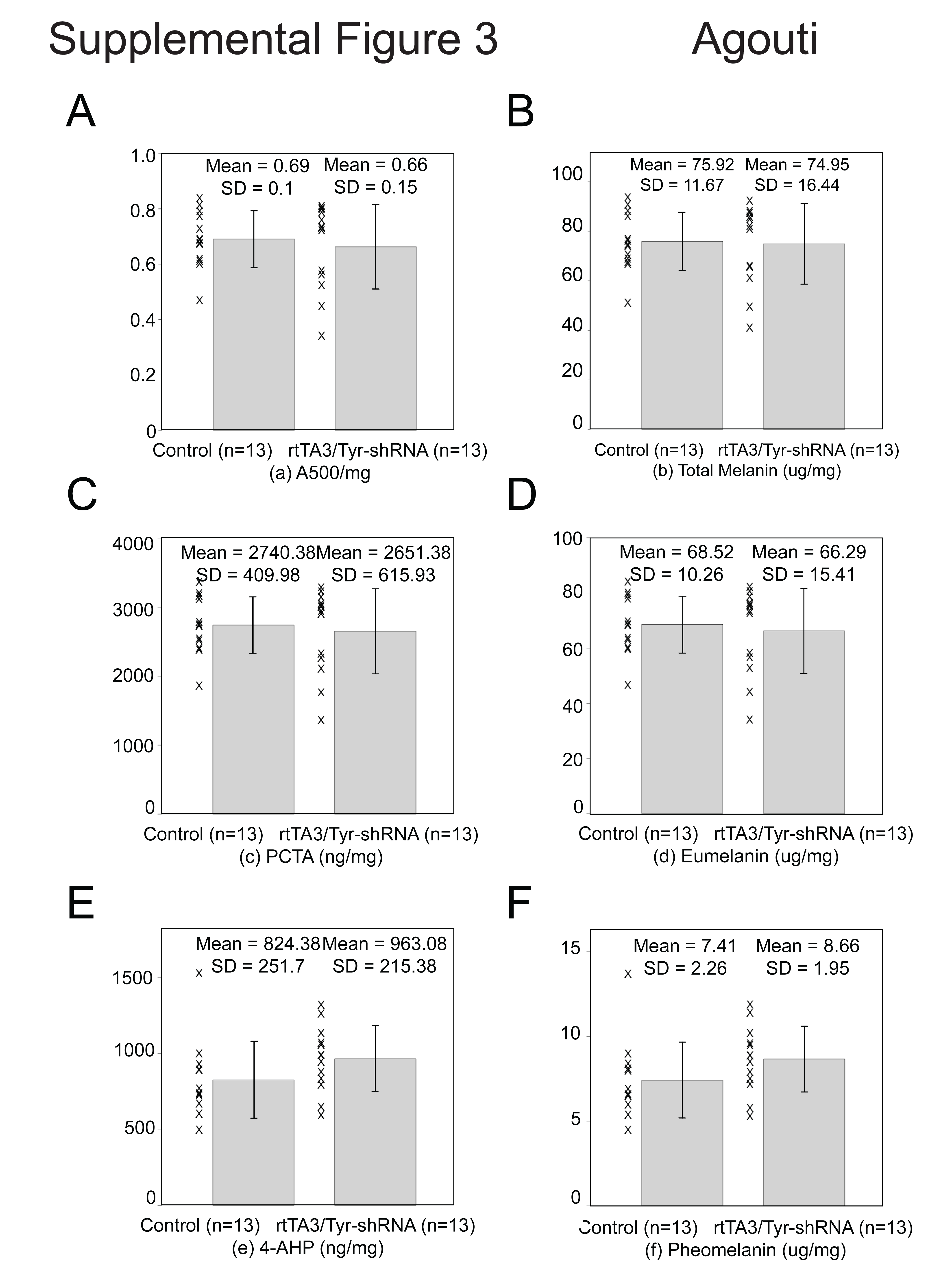

Supplement: S3 Fig — Bar plots depict the arithmetic mean and (standard deviation) for the rtTA3/Tyr-shRNA and control groups (actual numeric values given at the top of each subfigure). Dorsal hairs of agouti mice on continuous doxycycline treatment were shaved and processed for chemical analysis of eumelanin and of pheomelanin based upon their specific degradation products, pyrrole-2,3,5-tricarboxylic acid (PCTA) and 4-amino-3-hydroxyphenylalanine (4-AHP), respectively as described in Table 1. 13 control (Tyr-shRNA only or rtTA driver only) and 13 Tyr-knockdown mice were analyzed. ‘X’ symbols on the left of each bar represent the actual data observed for each group. (TIF) [file pone.0143702.s003.tif]

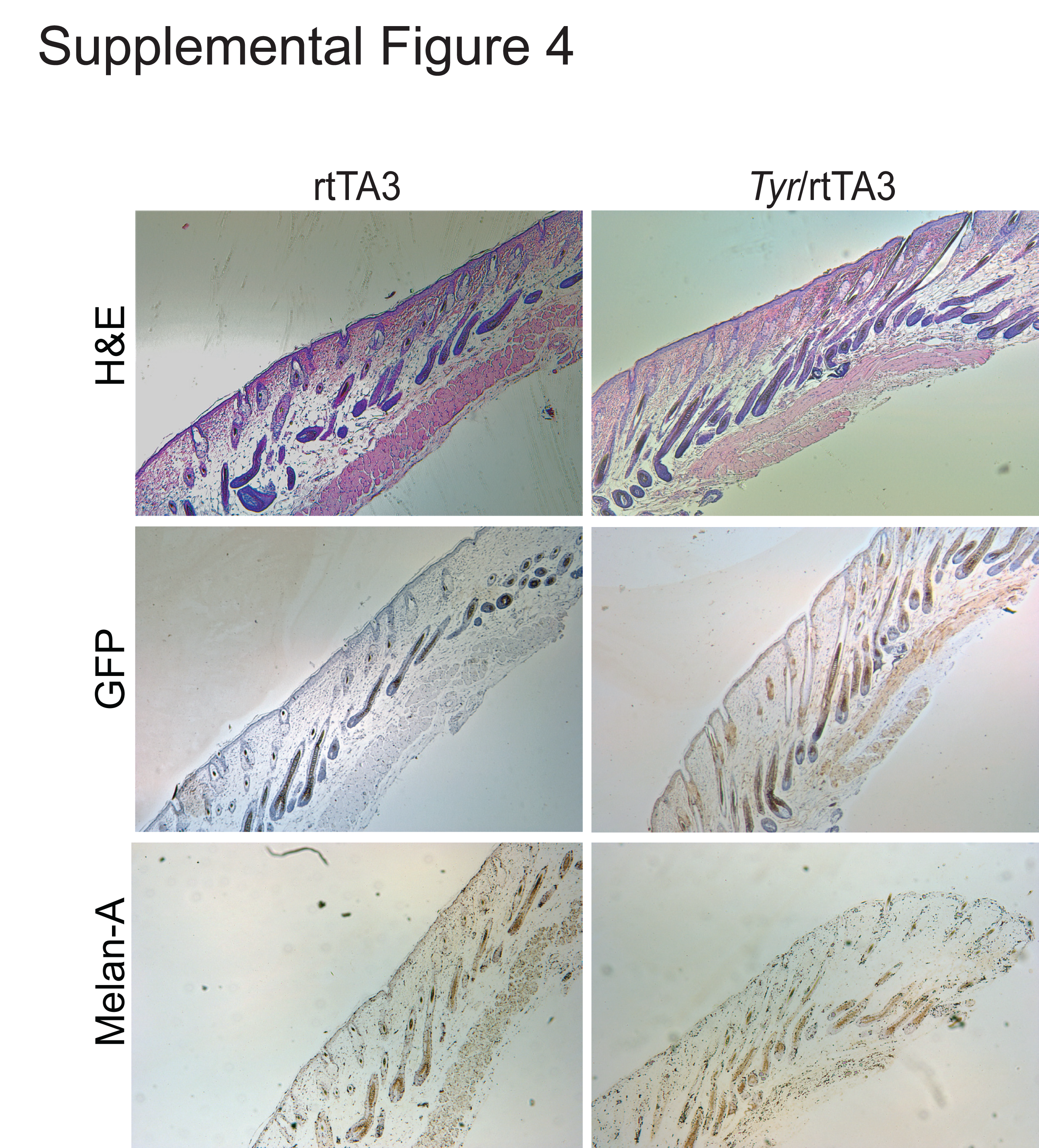

Supplement: S4 Fig — Four-mm skin punch biopsies taken from the indicated mice at P50 were formalin fixed, dehydrated, and paraffin embedded. Seven-μm thick sections of the skin were cut and stained for hematoxylin and eosin (top row), immunostained for GFP (middle row) and immunostained for Melan-A (bottom row). (TIF) [file pone.0143702.s004.tif]

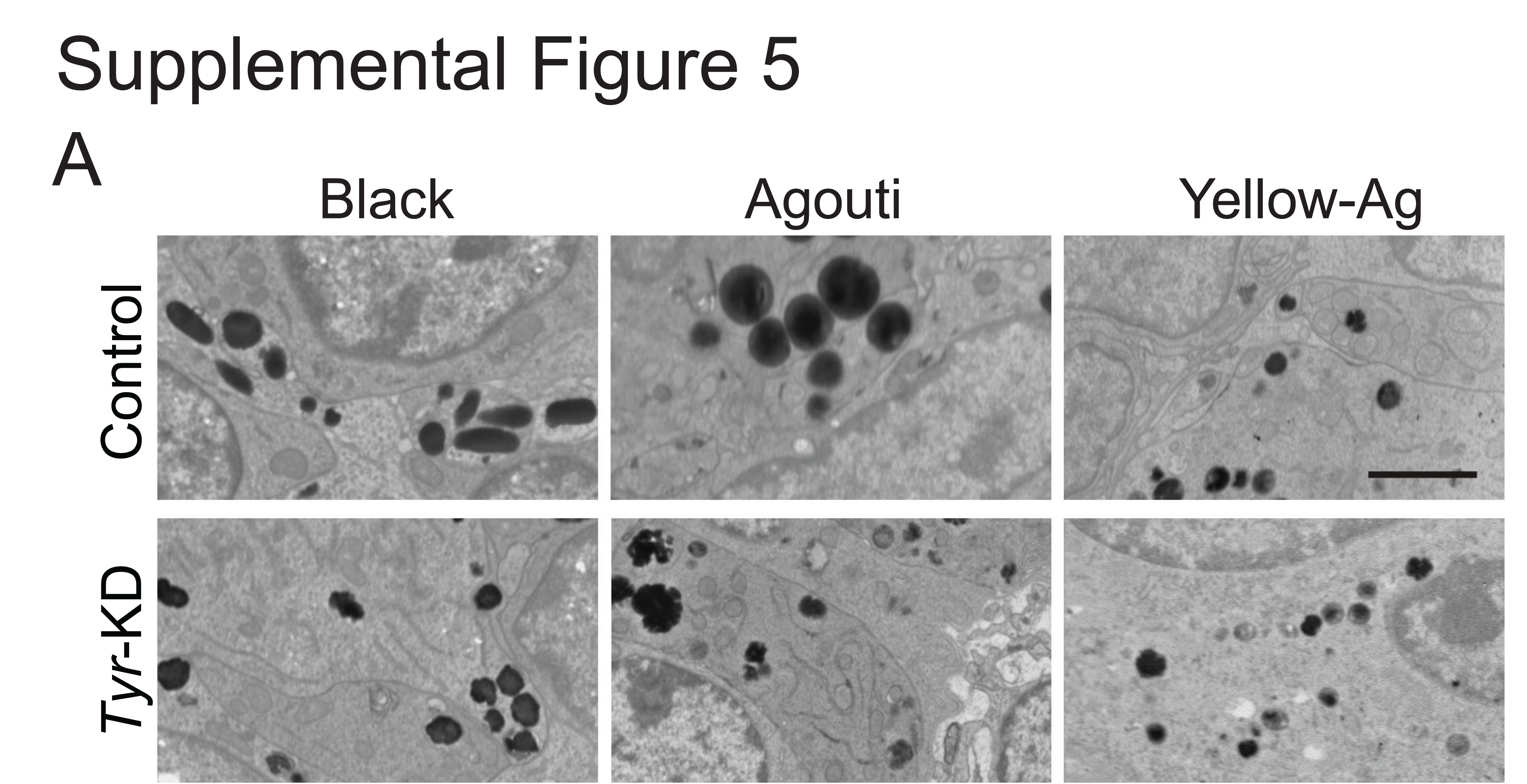

Supplement: S5 Fig — Fresh whole mouse skin was excised from Tyr-knockdown mice and corresponding control mice using a four-mm round punch biopsy and fixed in Karnovsky’s fixative before transmission electron microscopy analysis. Melanosomes within the dendrites of the melanocyte were then evaluated for ultrastructural morphology. Images are representative of 15 melanocytes from 2 mice per group. Bar = 2.5 microns. (TIF) [file pone.0143702.s005.tif]

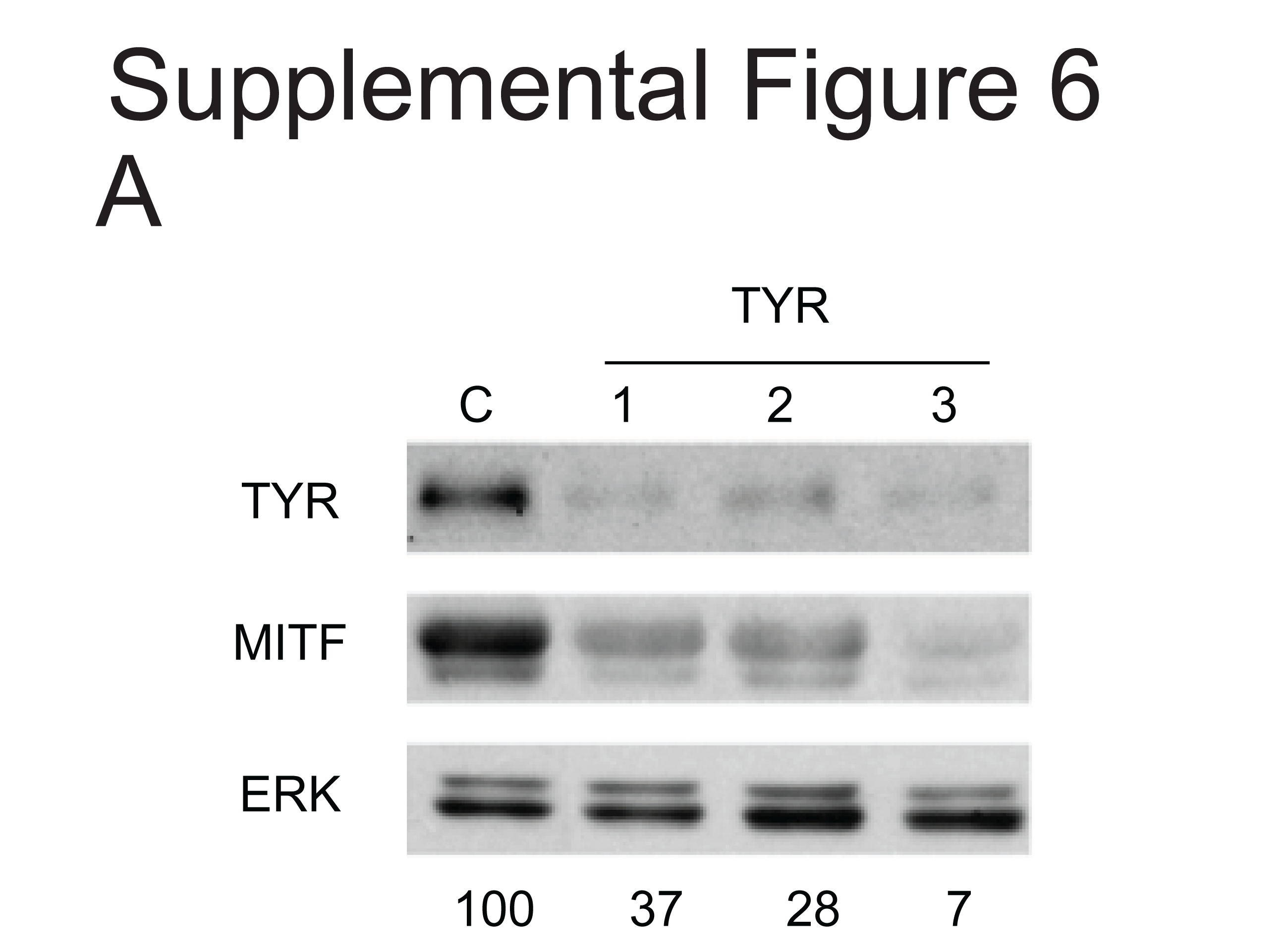

Supplement: S6 Fig — The impact of multiple independent siRNAs (1, 2, 3) targeting TYR, on TYR and MITF protein accumulation was assessed by immunoblot and compared to a control, mismatch siRNA (C). The impact of the given siRNAs on the protein accumulation of TYR and MITF was quantitated by densitometry (numbers below the corresponding blots). (TIF) [file pone.0143702.s006.tif]
