# Supplementary material for: Tyrosinase Depletion Prevents the Maturation of Melanosomes in the Mouse Hair Follicle
Source: PLoS One. 2015 Nov 30;10(11):e0143702. doi: 10.1371/journal.pone.0143702 (PMC4664286; doi:10.1371/journal.pone.0143702)
Supplement: S1 Table — Fresh whole mouse skin was excised from the indicated mice using a four-mm round punch biopsy and fixed in Karnovsky’s fixative before electron microscopy analysis. The number of melanosomes per area for 15 melanocytes from 2 mice per mouse coat color and genotype were determined. (DOC) [file pone.0143702.s007.doc]

| Mouse Coat Color and Genotype | Number of melanosomes/100 micron^2^ | *p*-value |
| --- | --- | --- |
| Black *Tyr*-shRNA control | 113.9 +15.4 | 0.24 |
| Black *Tyr*-knockdown | 108.9 +12.4 |  |
| Agouti  *Tyr*-shRNA control | 103.9 +7.5 | 0.29 |
| Agouti  *Tyr*-knockdown | 105.9 +10.9 |  |
| Yellow agouti  *Tyr*-shRNA control | 105.9 +10.9 | 0.43 |
| Yellow agouti  *Tyr*-knockdown | 94.6 +10.7 |  |
