# Supplementary material for: Tyrosinase Depletion Prevents the Maturation of Melanosomes in the Mouse Hair Follicle
Source: PLoS One. 2015 Nov 30;10(11):e0143702. doi: 10.1371/journal.pone.0143702 (PMC4664286; doi:10.1371/journal.pone.0143702)
Supplement: S2 Table — IHC-P = immunohistochemistry, paraffin-embedded tissue. WB = Western blot (DOC) [file pone.0143702.s008.doc]

| **Antibody** | **Host** | **Application** | **Dilution** | **Company** | **Catalog No.** |
| --- | --- | --- | --- | --- | --- |
| polyclonal anti-GFP | Rabbit | IHC-P | 1:1000 | Cell Signaling | 2555 |
| anti-rabbit IgG (biotinylated) | Goat | IHC-P | 1:500 | Vector Labs | BA-1000 |
| monoclonal anti-Melan-A | Mouse | IHC-P | 1:50 | Santa Cruz | sc-20032 |
| anti-mouse IgG (biotinylated) | Horse | IHC-P | 1:200 | Vector Labs | BA-2001 |
| polyclonal anti-TYR | Goat | WB | 1:200 | Santa Cruz | sc-7834 |
| monoclonal anti-GFP | Mouse | WB | 1:1000 | Cell Signaling | 2955 |
| monoclonal anti-β-actin | Rabbit | WB | 1:1000 | Cell Signaling | 4970 |
| anti-rabbit IgG, HRP-linked | Goat | WB | 1:5000 | Cell Signaling | 7074 |
| anti-mouse IgG, HRP-linked | Horse | WB | 1:5000 | Cell Signaling | 7076 |
| anti-goat IgG, HRP-linked | Rabbit | WB | 1:1000 | Santa Cruz | sc-2768 |
