# Supplementary material for: Tyrosinase Depletion Prevents the Maturation of Melanosomes in the Mouse Hair Follicle
Source: PLoS One. 2015 Nov 30;10(11):e0143702. doi: 10.1371/journal.pone.0143702 (PMC4664286; doi:10.1371/journal.pone.0143702)
Supplement: S3 Table — (DOC) [file pone.0143702.s009.doc]

| **Genotyping Primers** | **Sequence, 5’ to 3’** |
| --- | --- |
| Rosa26-rtTA2 common reverse | AAAGTCGCTCTGAGTTGTTAT |
| Rosa26-rtTA2 transgene forward | GCGAAGAGTTTGTCCTCAACC |
| Rosa26-rtTA2 wild-type forward | GGAGCGGGAGAAATGGATATG |
| CAG-rtTA3 common reverse | CGAAACTCTGGTTGACATG |
| CAG-rtTA3 transgene forward | CTGCTGTCCATTCCTTATTC |
| CAG-rtTA3 wild-type forward | TGCCTATCATGTTGTCAAA |
| RBG-reverse 1 | GAAAGAACAATCAAGGGTCC |
| RGB-reverse 2 | CACCCTGAAAACTTTGCCCC |
| *Col1a1*-forward | AATCATCCCAGGTGCACAGCATTGCGG |
| *Col1a1*-reverse | CTTTGAGGGCTCATGAACCTCCCAGG |
| SAdpA-reverse | ATCAAGGAAACCCTGGACTACTGCG |
| *Tyr*-shRNA forward 1 | AAGCCACAGATGTATGATCTGCTA |
| *Tyr*-shRNA forward 2 | GCCACAGATGTATGATCTGCTAC |
| *Luc*-shRNA forward | GATGTATTAATCAGAGACTTC |
| *Luc*-shRNA reverse | CACCCTGAAAACTTTGCCCC |
