# Supplementary material for: Tyrosinase Depletion Prevents the Maturation of Melanosomes in the Mouse Hair Follicle
Source: PLoS One. 2015 Nov 30;10(11):e0143702. doi: 10.1371/journal.pone.0143702 (PMC4664286; doi:10.1371/journal.pone.0143702)
Supplement: S4 Table — A—agouti, chromosome 2. B—tyrosinase related protein 1 (Tyrp1), chromosome 4. C—Tyrosinase (Tyr), chromosome 7. p—oculocutaneous albinism II (Oca2), aka pink-eyed dilution, chromosome 7. (DOCX) [file pone.0143702.s010.docx]

|  | Genotype at locus | | | |
| --- | --- | --- | --- | --- |
| Strain | *agouti* | *Tyrp1* | *Tyr* | *Oca2* |
| C57BL/6J | *a/a* | *+/+* | *+/+* | *+/+* |
| B6.Cg-Ay/J | *A^y^/a* | *+/+* | *+/+* | *+/+* |
| KH2 ES cells (129S4 x C57BL/6J F1) | *A^w^/a* | *+/+* | *+/+* | *+/+* |
| B6N.FVB(Cg)-Tg(CAG-rtTA3)4288Slowe/J | a/a | *+/+* | *+/+* | *+/+* |
| 129S1/SvImJ | *A^w^/A^w^* | *+/+* | *+/+* | *+/+* |
